# Analysis of the quality of tunnel roof topography by automatic cutting control under the coupling of multiple factors

**DOI:** 10.1371/journal.pone.0299805

**Published:** 2024-03-21

**Authors:** Jinnan Lu, Bo Li, Yun Zhu, Miao Xie, Qingshuang Meng, Zhixiang Liu, Yufeng Dong

**Affiliations:** 1 School of Mechanical Engineering, Liaoning Technical University, Fuxin, China; 2 Faculty of Electrical and Control Engineering, Liaoning Technical University, Huludao, China; 3 Key Laboratory of High Performance Manufacturing for Aero Engine, Ministry of Industry and Information Technology, Northwestern Polytechnical University, Xi’an, China; 4 Research Institute of Mineral Resources Development and Utilization Technology and Equipment, Liaoning Technical University, Fuxin, China; University of Vigo, SPAIN

## Abstract

The automatic cutting of coal and rock surface morphology modeling based on the actual geological environment of coal mine underground excavation and mining is of great significance for improving the surface quality of coal and rock after cutting and enhancing the safety and stability of advanced support. To this end, using the principle of coordinate transformation, the kinematic trajectory of the cutting head of the tunneling machine is established, and the contour morphology of the cutting head under variable cutting technology is obtained. Then, based on the regenerative vibration theory of the cutting head, a dynamic model of the cutting head coal wall is established, and the coordinate relationship of the cutting head in the tunnel coordinate system under vibration induction is analyzed. Based on fractal theory and Z-MAP method, a simulation method for the surface morphology of coal and rock after cutting is proposed, which is driven by the cutting trajectory Under the coupling effect of cutting vibration induction and random fragmentation of coal and rock, simulation of the surface morphology of comprehensive excavation tunnels was conducted, and relevant experiments were conducted to verify the results. A 1:3 similarity experimental model of EBZ160 tunneling machine was used to build a cutting head coal and rock system cutting experimental platform for comparative experiments of cutting morphology. Furthermore, statistical methods were used to compare and evaluate the simulated roof with the actual roof. The results show that the relative errors between the maximum range of peaks and valleys, the peak skewness coefficient of height standard deviation, and the kurtosis coefficient of the actual roof are 1.3%, 24.5%, 16%, and 2.9%, respectively. Overall, this indicates that the surface morphology distribution characteristics of the simulated roof and the actual roof are similar, verifying the effectiveness of the modeling and simulation method proposed in this paper, and providing theoretical support for the design and optimization of advanced support in the future.

## Introduction

In the intelligent excavation process, the excavation machine automatically cuts according to the predetermined cutting trajectory during the cutting process. Due to differences in the shape of the cutting head, cutting process, and geological conditions of the roof, the cut roof presents different rough morphology characteristics. The surface morphology of coal and rock after cutting is closely related to the contact state, contact form, friction, vibration of the advanced support roof. Therefore, determining the surface morphology of coal and rock after cutting is crucial for achieving timely and effective advanced support in coal mines.

When automatic cutting is carried out by a longitudinal axis tunneling machine in a coal mine, the most critical factors affecting the surface morphology characteristics of the tunnel in the early stage of formation are the movement trajectory of the cutting head and the external parameters of the cutting head [1].

Zhang et al. [2] studied the cutting trajectory during the cutting process, the relationship between the plastic deformation of the surrounding rock after cutting and the surface roughness of the roadway. Liu et al. [3] studied the irregular morphology of the surrounding rock in the tunnel space after cutting based on the excavation process of the excavation machine and the external dimensions of the cutting head, and proposed an evaluation method for the roughness of the roof of the excavation working face. Zhang et al. [4] studied the distribution and height characteristics of the roof of the excavation face after cutting, and analyzed the influence of the geometric motion of the cutting head on it. Liu et al. [5] studied the morphological characteristics of the rough surface of coal and rock tunnels, and conducted in-depth research on the contour forming mechanism of the tunnel cut by the cutting head of the tunneling machine. The influence law between the cutting process parameters and the surface roughness of the cut formed tunnel was obtained.

Therefore, many experts and scholars have begun to study the use of fractal theory as a quantitative evaluation method for the roughness of structural joints. Some scholars have proposed the code ruler method and box dimension method based on self-similarity characteristics [6, 7] and derived the fractal dimension expression of the joint profile of the structural plane, obtaining the relationship between the fractal dimension and the joint roughness coefficient. However, Russ [8]. found through analysis of natural rock joint profiles that it exhibits more affine characteristics rather than self-similarity. Therefore, subsequent scholars have improved their research methods for self-similarity characteristics. Xia et al. [9] have modified the above methods and obtained a fractal dimension derivation method based on self-affine joint profiles Some researchers, such as Sun [10] Fardin [11] and Xie [12] have directly quantified the joint roughness coefficient of natural surrounding rocks through self-affine fractal. Research has shown that the quantification method obtained through self-affine fractal is clearly the quantification result obtained directly through parameter estimation.

Given the complex and rough morphology of surrounding rock, many scholars have incorporated different factors into it to deeply explore the roughness of rock joint surfaces in nature. Therefore, Wang et al. [13] and Ding et al. [14] combined fractal dimension D with amplitude parameter A to more accurately reflect the roughness of rock masses in nature.

In summary, it can be seen that domestic and foreign experts and scholars have conducted extensive research on the quantitative methods of the roughness of tunnel surrounding rock through a combination of theoretical and experimental research. The research results show that the quantitative characterization of the roughness of naturally formed coal rock joint surfaces is usually determined by objective factors such as roughness amplitude, roughness slope or spatial changes. However, the roof of the cut and formed roadway is often influenced by the coupling dynamics between the cutting head morphology and the cutting process. Currently, there is a lack of a unified description method for the coal rock morphology characteristics after cutting under the influence of various factors mentioned above.

**Fig 1:**
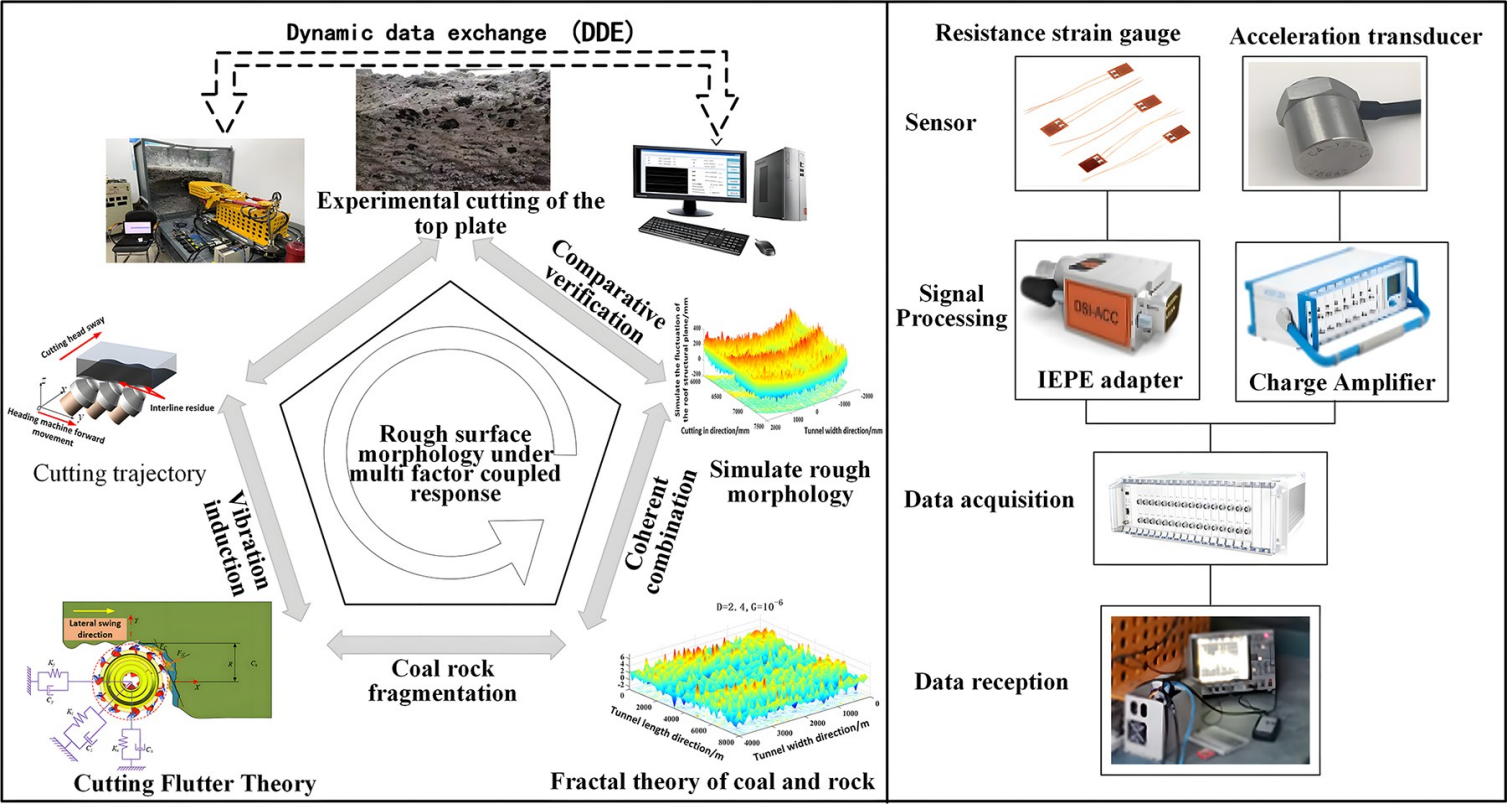
Modeling method for roadway roof roughness based on multi-source coupling.

In this article, simulation modeling research is conducted on the morphology characteristics of the roof in the empty roof area between the permanent support and the excavation face after cutting from three perspectives: cutting trajectory, cutting vibration, and fractal characteristics of the coal and rock after cutting. Finally, combined with the longitudinal and transverse morphology of the roof obtained above, as well as the fracture of the roof after cutting, a modeling method for the rough surface morphology of the comprehensive excavation roadway under the coupling of multiple factors is considered. This provides a theoretical basis for the three-dimensional reconstruction of the roof after automatic cutting, and the modeling method for the roughness of the roadway roof is shown in Fig 1.

This article is based on the actual rough roadway roof in coal mine working faces. Through analyzing the cutting morphology under the longitudinal feed of the cutting head, the correction of the cutting head’s posture under induction, and the random collapse of the roadway roof random morphology described by fractal theory, a comprehensive quantitative study of the roof roughness is carried out. And the extraction of actual roof morphology features was achieved through grayscale images of the roadway roof. The effectiveness of the proposed rough roof quantification evaluation method was demonstrated using statistical methods to evaluate the effectiveness of the simulation roof quantification method.

## Establishment of trajectory equation for cutting geometry

### Analysis of cutting motion of tunneling machine

In the process of studying the surface morphology of tunnels, building an accurate tunnel model and calculating the position changes of the cutting head are crucial for completing simulation tasks. How to find the intersection of the cutting head motion model and the tunnel model is key to the entire simulation work.

**Fig 2:**
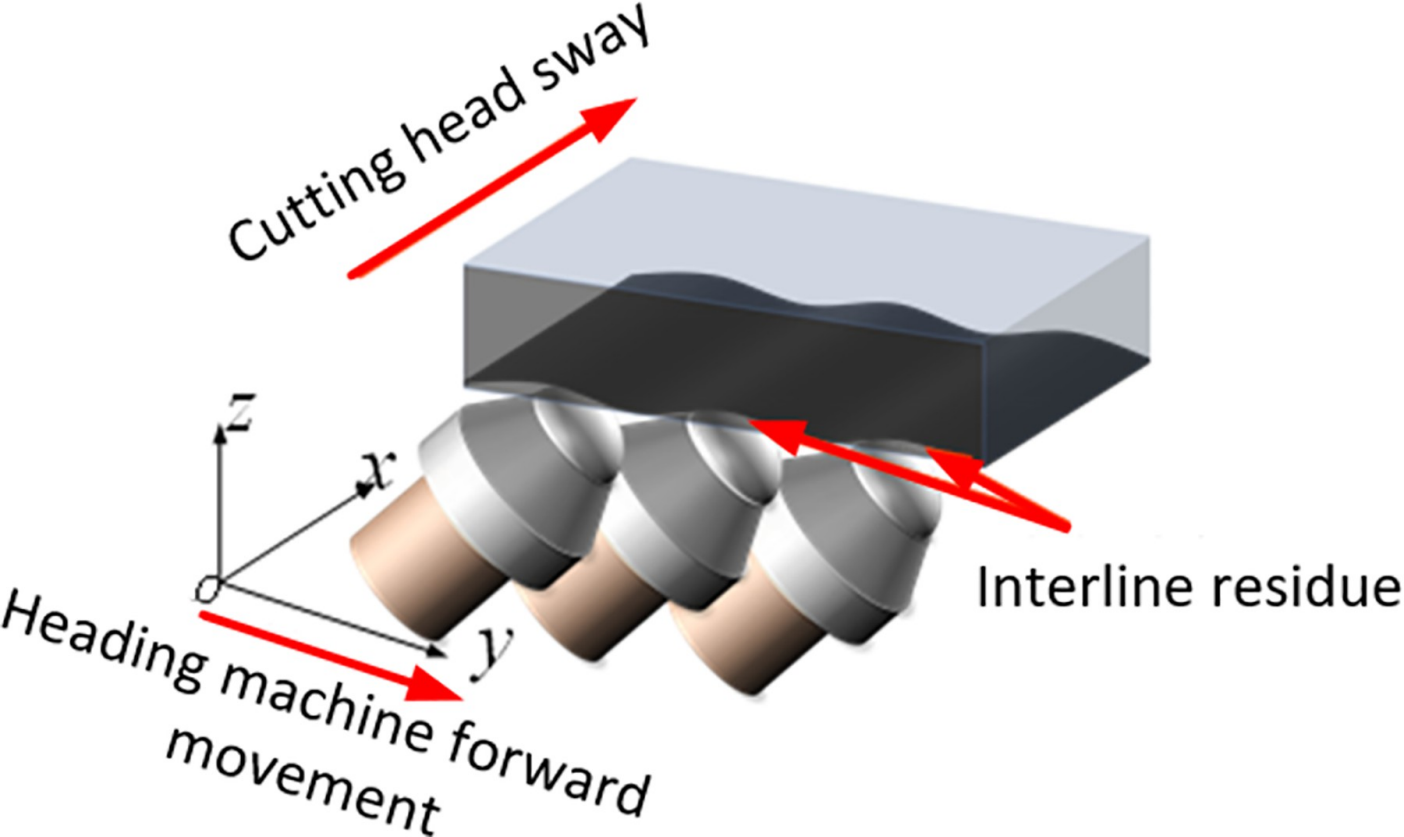
Topplate morphology after cutting without considering discrete cutting teeth.

The movement trajectory of the cutting head will directly affect the cutting morphology of the roadway roof, and different shapes of the roadway roof will lead to differences in the roughness of the roadway roof. The cutting process of the tunneling machine’s cutting head is similar to that of milling workpieces with a milling cutter. The cutting motion can be divided into lateral feed motion and main motion, that is, the rotation of the cutting head itself around the spindle. Therefore, under ideal conditions, without considering the discreteness of the cutting teeth, an ideal fanless residual surface can be obtained. As shown in Fig 2, at this point, the obtained surface is only generated by the inter row residue generated by the tunneling machine’s stepping.

When using traditional manual control to cut coal and rock in tunnels, it is often accompanied by repeated repair and repair, resulting in the loss of certain natural characteristics in the cutting morphology. In the intelligent excavation system, when the same cutting method is used for cutting coal and rock tunnels, the cutting head gradually cuts the coal and rock from the predetermined cutting trajectory from bottom to top, and the complex formation of the coal and rock after cutting is generated by the relative motion between the cutting head and the coal and rock. Therefore, when considering the complex morphology of the roadway roof after cutting, it is necessary to consider the cutting head morphology (cutting head contour shape, external dimensions) and the spatial position changes related to the cutting head during cutting operations, including the position of the tunneling machine, the lifting angle of the cutting part and other related factors [15, 16].

When simulating the shape of the roadway roof, we simplified the cutting process by omitting the process of cutting the coal and rock below the roof, while ignoring the role of distributed cutting teeth during the cutting process. It is assumed that the part of the roof swept by the cutting head of the tunneling machine completely falls off, and the process of sweeping the wall and roof is omitted in the cutting simulation. That is, after completing a lateral movement during the cutting process of the top plate, the tunneling machine moves forward one step.

Taking the 122304 transport trough working face of Zhamei Tiebei Coal Mine as an example, the working face is characterized by an east and west monoclinic structure, with little variation in the dip angle of the coal seam within the working face between 1°and 2°. The excavation roadway is located in the Yimin Formation, mainly composed of sandstone, siltstone, mudstone, carbonaceous mudstone, et al. The coal seam in the working face is fully developed. According to the analysis of geological drilling data and supplementary survey report data, there are no geological structures such as faults, folds, and magmatic intrusions developed in the 122304 working face. The attribute characteristics of the coal seam are shown in Table 1.

**Table 1:** Coal seam occurrence characteristics.

project	index	notes
average thickness of coal seam /m	3.0	
dip angle of coal seam /°	1~2	
coal seam hardnessf	2.5	low hardness and easy to break
coal seam bedding (degree of development)	more developed	
spontaneous ignition period /d	minimum spontaneous ignition period 41	type I spontaneous combustion coal seam
absolute gas emission / (m^3^/min)	0.224	
Coal dust explosion index /%	39.46	

### Establishment of cutting geometry motion trajectory equation

**Fig 3:**
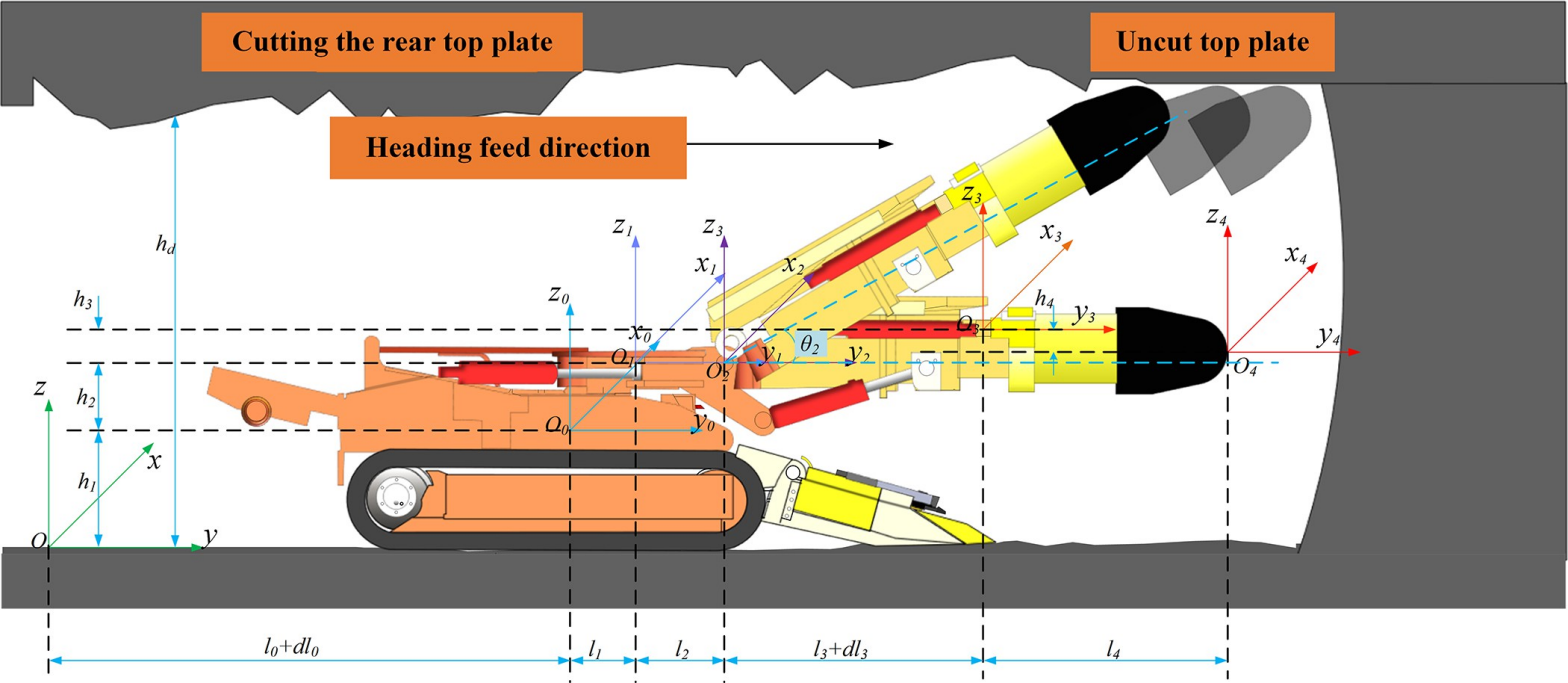
Spatial coordinate transformation during the operation of the tunneling machine.

As shown in Fig 3, establish the roadway reference coordinate system *OXYZ*, the center coordinate system of the entire tunneling machine *O*_*0*_*X*_*0*_*Y*_*0*_*Z*_*0*_, the rotary table rotation coordinate system *O*_*1*_*X*_*1*_*Y*_*1*_*Z*_*1*_, the cutting part lifting coordinate system *O*_*2*_*X*_*2*_*Y*_*2*_*Z*_*2*_, the cutting part stretching coordinate system *O*_*3*_*X*_*3*_*Y*_*3*_*Z*_*3*_, and the cutting head coordinate system *O*_*4*_*X*_*4*_*Y*_*4*_*Z*_*4*_. Due to the fact that the motion process of the entire tunneling machine is in series and also belongs to the open chain motion system. Therefore, the D-H method can be used to perform coordinate transformation calculation of the cutting head position and complete kinematic analysis. In the D-H analysis method, the rotation of the rotary table, the lifting and retraction of the cutting part, and the process of coordinate transformation are considered simultaneously. This is used to describe the spatial pose coordinates of the cutting head in motion compared to the actual position and attitude of the tunnel coordinate system. Furthermore, the transformation matrix between each coordinate system can be obtained:

$$\begin{array}{l} {\;}_{0}T=\left[\begin{array}{cccc} 1 & 0 & 0 & 0\\ 0 & 1 & 0 & l_{0}+(n-1)dl_{0}\\ 0 & 0 & 1 & h_{1}\\ 0 & 0 & 0 & 1 \end{array}\right]{\;}_{1}^{0}T={\left[\begin{array}{cccc} \cos(\theta_{0}+\varpi t) & -\sin(\theta_{0}+\varpi t) & 0 & 0\\ \sin(\theta_{0}+\varpi t) & \cos(\theta_{0}+\varpi t) & 0 & l_{1}\\ 0 & 0 & 1 & h_{2}\\ 0 & 0 & 0 & 1 \end{array}\right]}_{2}^{1}\\ {\;}_{2}^{1}T=\left[\begin{array}{cccc} 1 & 0 & 0 & 0\\ 0 & \cos\theta_{2} & -\sin\theta_{2} & l_{2}\\ 0 & \sin\theta_{2} & \cos\theta_{2} & 0\\ 0 & 0 & 0 & 1 \end{array}\right]{\;}_{3}^{2}T=\left[\begin{array}{cccc} 1 & 0 & 0 & 0\\ 0 & 1 & 0 & l_{3}+dl_{3}\\ 0 & 0 & 1 & h_{3}\\ 0 & 0 & 0 & 1 \end{array}\right]{\;}_{4}^{3}T=\left[\begin{array}{cccc} 1 & 0 & 0 & 0\\ 0 & 1 & 0 & l_{4}\\ 0 & 0 & 1 & -h_{4}\\ 0 & 0 & 0 & 1 \end{array}\right] \end{array}$$ (1)

Therefore, it is easy to obtain the homogeneous transformation matrix of the cutting head coordinate system in the tunnel coordinate system:

$${\;}_{4}T={\;}_{0}T{\;}_{1}^{0}T{\;}_{2}^{1}T{\;}_{3}^{2}T{\;}_{4}^{3}T=[\begin{array}{cccc} \cos(\theta_{0}+\omega t) & -\cos(\theta_{0}+\omega t)\sin(\theta_{0}+\omega t) & \sin{\left(\theta_{0}+\omega t\right)}_{1}\sin\theta_{2} & \left[\begin{array}{c} \left(h_{3}-h_{4})\sin{\left(\theta_{0}+\omega t\right)}_{1}\sin\theta_{2}-l_{2}\sin(\theta_{0}+\omega t\right)\\ -\cos\theta_{2}\sin(\theta_{0}+\omega t)(dl_{3}+l_{3}+l_{4}) \end{array}\right]\\ \sin{\left(\theta_{0}+\omega t\right)}_{1} & \cos{\left(\theta_{0}+\omega t\right)}_{1}\cos\theta_{2} & -\cos{\left(\theta_{0}+\omega t\right)}_{1}\sin\theta_{2} & \left[\begin{array}{c} (n-1)dl_{0}+l_{0}+l_{1}+l_{2}\cos(\theta_{0}+\omega t)+\\ \cos(\theta_{0}+\omega t)\cos\theta_{2}(dl_{3}+l_{3}+l_{4})\\ -(h_{3}-h_{4})\cos{\left(\theta_{0}+\omega t\right)}_{1}\sin\theta_{2} \end{array}\right]\\ 0 & \sin\theta_{2} & \cos\theta_{2} & h_{1}+h_{2}+\sin\theta_{2}(dl_{3}+l_{3}+l_{4})+\cos\theta_{2}\\ 0 & 0 & 0 & 1 \end{array}]$$ (2)

In the above equation, *l*_*i*_ (*i* = 0~4 represents the horizontal distance in different coordinate systems) (mm), while *h*_*i*_ (*i* = 1~4) represents the vertical distance in different coordinate systems (mm). And *d*_*l0*_ represents the cutting feed amount between each step of the tunneling machine (mm); *n* represents the number of cycles of cutting by the tunneling machine, *n = 1*, *2*, *3*; *d*_*l3*_ represents the stretching displacement of the tunneling machine during the cutting process (mm); *h*_*d*_ represents the height of the cut roadway (mm); *θ*_*0*_ represents the initial horizontal angle of the rotary table when cutting the top plate (°); *ω*represents the swinging speed of the cutting head during roof cutting (rad/s).

If the coordinates of a point are defined as: ${\;}^{4}p={\left[\begin{array}{cccc} x_{j} & y_{j} & z_{j} & 1 \end{array}\right]}^{T}$, its position in the tunnel coordinate system can be calculated through the coordinate transformation matrix, thus achieving accurate positioning of it. The results are as follows:

$$\begin{array}{l} p={\;}_{4}T^{4}p={\left[\begin{array}{cccc} x_{h} & y_{h} & z_{h} & 1 \end{array}\right]}^{T}\\ =\left[\begin{array}{c} \cos\theta_{1}-\sin\theta_{1}(l_{2}+{\mathrm{dl}}_{3}\cos\theta_{2}+l_{3}\cos\theta_{2}+l_{4}\cos\theta_{2}-h_{3}\sin\theta_{2}+h_{4}\sin\theta_{2})+z_{j}\sin\theta_{1}\sin\theta_{2}-y_{j}\cos\theta_{2}\sin\theta_{1}\\ {\mathrm{dl}}_{0}+l_{0}+l_{1}+x_{j}\sin\theta_{1}+l_{2}\cos\theta_{1}+\cos\theta_{1}\cos\theta_{2}({\mathrm{dl}}_{3}+l_{3}+l_{4}+y_{j})-(h_{3}-h_{4}-z_{j})\cos\theta_{1}\sin\theta_{2}\\ h_{1}+h_{2}+z_{j}\cos\theta_{2}+(dl_{3}+l_{3}+y_{j}+l_{4})\sin\theta_{2}+(h_{3}-h_{4})\cos\theta_{2}+l_{4}\sin\theta_{2}\\ 1 \end{array}\right] \end{array}$$ (3)

**Fig 4:**
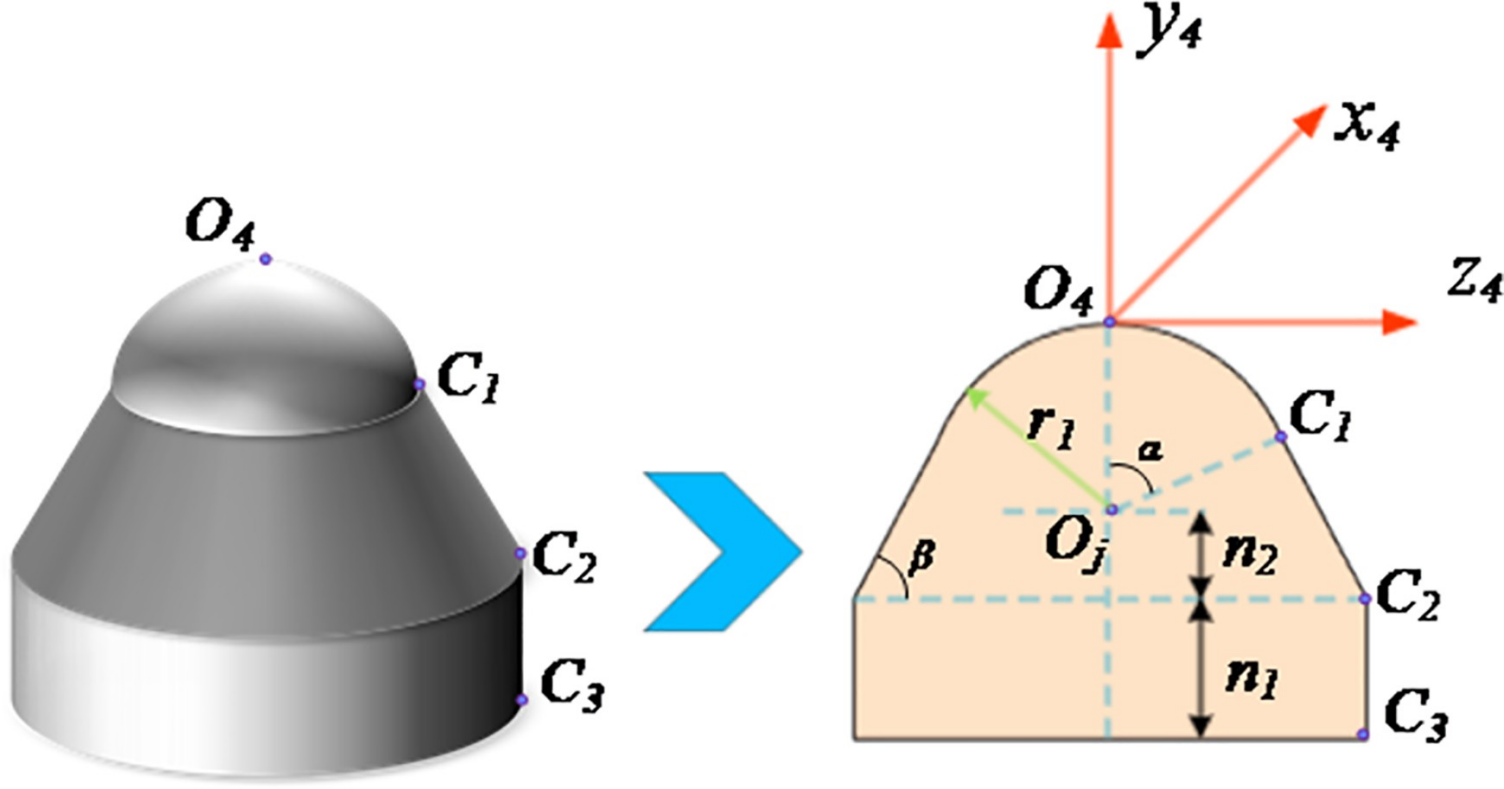
Cutting head type and coordinate definition.

At the same time, due to the different contour shapes of the cutting heads of the tunneling machine, the residual shapes between the rows generated after cutting are different. Currently, the three common cutting head forms of the tunneling machine are: "ball crown+cylinder" type, "ball crown+round table" type and "ball crown+round table+cylinder" type. Due to the most widespread application of the "ball crown+round table+cylinder" type in tunneling machines. This article selects the "spherical crown+circular table+cylindrical" cutting head to construct the cutting surface contour model. Through the above kinematic analysis, we can combine the cutting matrix coordinate system of the tunneling machine, the tunnel spatial coordinate system, and the coordinate system of each mechanism of the tunneling machine to convert the spatial positions of the cutting key nodes. The shape of the cutting head and the spatial coordinates of key nodes *O*_*4*_, *O*_*j*_, *C*_*1*_, *C*_*2*_, *C*_*3*_ (j = 1, 2, 3, 4) in the cutting head coordinate system are shown in Fig 4.

As shown in Fig 4, *α* is the half included angle of the arc of the cutting head ball crown, *β* is the cone angle of the cutting head cone, *r*_*1*_ is the radius of the cutting head spherical crown, *n*_*1*_ is the height of the cylindrical part of the cutting head, and *n*_*2*_ is the height of the cutting head cone. Through the expression in the above figure, the coordinates of each key node in the cutting head coordinate system can be obtained as follows:

$$\{\begin{array}{c} {\;}^{4}p_{O_{4}}={\left[\begin{array}{llll} 0 & 0 & 0 & 1 \end{array}\right]}^{T}\\ {\;}^{4}p_{O_{j}}={\left[\begin{array}{llll} 0 & -r_{1} & 0 & 1 \end{array}\right]}^{T}\\ {\;}^{4}p_{C_{1}}={\left[\begin{array}{llll} 0 & -r_{1}(1-\cos\alpha ) & r_{1}\sin\alpha & 1 \end{array}\right]}^{T}\\ {\;}^{4}p_{C_{2}}={\left[\begin{array}{llll} 0 & -r_{1}-n_{1} & r_{1}\sin\alpha +(n_{1}+r_{1}\cos\alpha )/\tan\beta & 1 \end{array}\right]}^{T}\\ {\;}^{4}p_{C_{3}}={[\begin{array}{llll} 0 & -r_{1}-n_{1}-n_{2} & r_{1}\sin\alpha +n_{2}/\tan\beta & 1] \end{array}}^{T} \end{array}$$ (4)

And through the coordinate transformation matrix $p{=}_{4}T^{4}p={\left[x_{p}\;y_{p}\;z_{p}\;1\right]}^{T}$, the coordinates of each point relative to the lane to the coordinate system can be obtained.


$$\left\{\begin{array}{c} p_{O_{4}}{=}_{4}T^{4}p_{O_{4}}={\left[\begin{array}{llll} 0 & 0 & 0 & 1 \end{array}\right]}^{T}\\ p_{O_{j}}{=}_{4}T^{4}p_{O_{j}}={\left[\begin{array}{llll} X_{O_{j}} & Y_{O_{j}} & Z_{O_{j}} & 1 \end{array}\right]}^{T}\\ p_{C_{1}}{=}_{4}T^{4}p_{C_{1}}={\left[\begin{array}{llll} X_{C_{1}} & Y_{C_{1}} & Z_{C_{1}} & 1 \end{array}\right]}^{T}\\ p_{C_{2}}{=}_{4}T^{4}p_{C_{2}}={\left[\begin{array}{llll} X_{C_{2}} & Y_{C_{2}} & Z_{C_{2}} & 1 \end{array}\right]}^{T}\\ p_{C_{3}}{=}_{4}T^{4}p_{C_{3}}={\left[\begin{array}{llll} X_{C_{3}} & Y_{C_{3}} & Z_{C_{3}} & 1 \end{array}\right]}^{T} \end{array}\right]$$ (5)

Therefore, due to the overall rotary structure of the cutting head, the overall outer contour of the cutting head can be represented by an equation composed of key points. Therefore, by using the coordinates of key nodes *O’*_*4*_, *O*_*j*_, *C*_*1*_, *C*_*2*_ and *C*_*3*_ in the tunnel coordinate system, the representation method of arc segment *O*_*4*_*C*_*1*_, straight segment *C*_*1*_*C*_*2*_ and straight segment *C*_*2*_*C*_*3*_ in the tunnel coordinate system can be obtained as follows:

$$\begin{array}{c} O_{4}C_{1}:Z=Z_{O_{j}}+r_{1}\sin(\arccos\frac{Y-Y_{O_{j}}}{r_{1}})\\ Y_{O_{j}}+r_{1}\cos\theta_{2}⩽Y⩽Y_{O_{j}}+r_{1}\cos(\theta_{2}+\alpha )\\ C_{1}C_{2}:Z=\frac{Z_{C_{2}}-Z_{C_{1}}}{Y_{C_{2}}-Y_{C_{1}}}(Y-Y_{C_{2}})+Z_{C_{1}}\\ Y_{C_{1}}⩽Y⩽Y_{C_{2}}\\ C_{2}C_{3}:Z=\frac{Z_{C_{3}}-Z_{C_{2}}}{Y_{C_{3}}-Y_{C_{2}}}(Y-Y_{C_{3}})+Z_{C_{2}}\\ Y_{C_{2}}⩽Y⩽Y_{C_{3}} \end{array})$$ (6)

**Fig 5:**
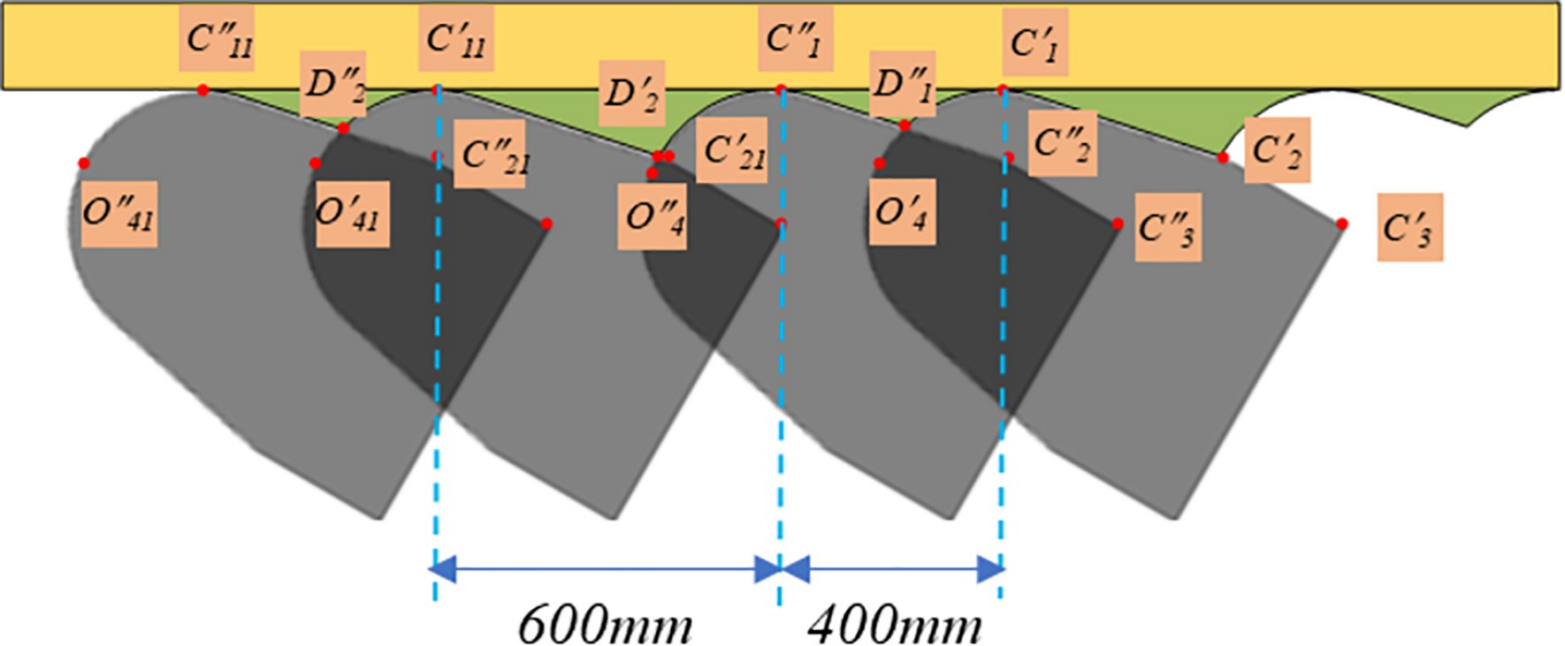
Cutting head circularly cuts the intersection point of outer contour.

Taking the excavation operation regulations of the 122304 working face of Tiebei Coal Mine in Zhamei as an example, the excavation machine has a feed cycle progress of 1000mm, divided into two cuts, the initial feed of 400mm and the second feed of 600mm to complete the remaining cuts. Therefore, during the process of one cycle progress, the outer contour of the cutting head obtained from the two cuts intersects at *D”*_*1*_. Among them, *D”*_*1*_ is located in the arc *O’*_*4*_*C’*_*1*_ segment of the previous feed. At the same time in the straight line *C”*_*1*_*C”*_*2*_ segment of the subsequent feed. Within the two adjacent cycles, the intersection point *D’*_*2*_ is also generated between the last feed of the first cycle and the outer contour line of the cutting head of the first feed of the second cycle. The intersection point is located in the arc *O”*_*4*_*C”*_*1*_ segment of the first cycle and the straight line *C’*_*11*_*C’*_*21*_ segment of the second cycle. Therefore, the inter row residue generated by the feeding of the tunneling machine can be considered as the alternating superposition of the *C”*_*1*_*D”*_*1*_*C’*_*1*_ area and the *C’*_*11*_*D’*_*2*_*C”*_*1*_ area. As shown in Fig 5, by solving the above equation, the description equation of the intersection coordinates and the outer contour can be obtained, as shown in Formula (7).


$$\begin{array}{l} C_{1}^{\prime \prime }D_{1}^{\prime \prime }:Z=\frac{Z_{C_{1}^{\prime \prime }}-Z_{D_{1}^{\prime \prime }}}{Y_{C_{1}^{\prime \prime }}-Y_{D_{1}^{\prime \prime }}}(Y-Y_{C^{\prime \prime }})+Z_{C^{\prime \prime }},Y_{C_{1}^{\prime \prime }}⩽Y⩽Y_{D_{1}^{\prime \prime }}\\ D_{1}^{\prime \prime }C_{1}^{\prime }:Z=Z_{O_{j}^{\prime }}+r_{1}\sin(\arccos\frac{Y-Y_{O_{j}^{\prime }}}{r_{1}}),Y_{C_{1}^{\prime }}⩽Y⩽Y_{D_{1}^{\prime }}\\ C_{11}^{\prime }D_{2}^{\prime }:Z=\frac{Z_{C_{11}^{\prime }}-Z_{D_{2}^{\prime }}}{Y_{C_{11}^{\prime }}-Y_{D_{2}^{\prime }}}(Y-Y_{C_{11}^{\prime }})+Z_{C_{11}^{\prime }},Y_{C_{11}^{\prime }}⩽Y⩽Y_{D_{2}^{\prime }}\\ D_{2}^{\prime }C_{11}^{\prime }:Z=Z_{O_{j}^{\prime }}+r_{1}\sin(\arccos\frac{Y-Y_{O^{\prime \prime }}}{r_{1}}),Y_{D_{2}^{\prime }}⩽Y⩽Y_{C_{11}^{\prime }} \end{array}$$ (7)

For the feed rate in the above contour equation, it can be expressed as: $f=ndl_{01}+(n-1)dl_{02}+\varphi_{i}dl_{02}$, where *dl*_*01*_ = 400mm; *dl*_*02*_ = 600mm; *φ*_*i*_ = 1-*φ*_*i-1*_, *φ*_*1*_ = 0.

Therefore, through the above equation, the outer contour of the cutting head movement during the formation of coal and rock in the cutting head can be completed using MATLAB software.

### Overview of Z-map algorithm

**Fig 6:**
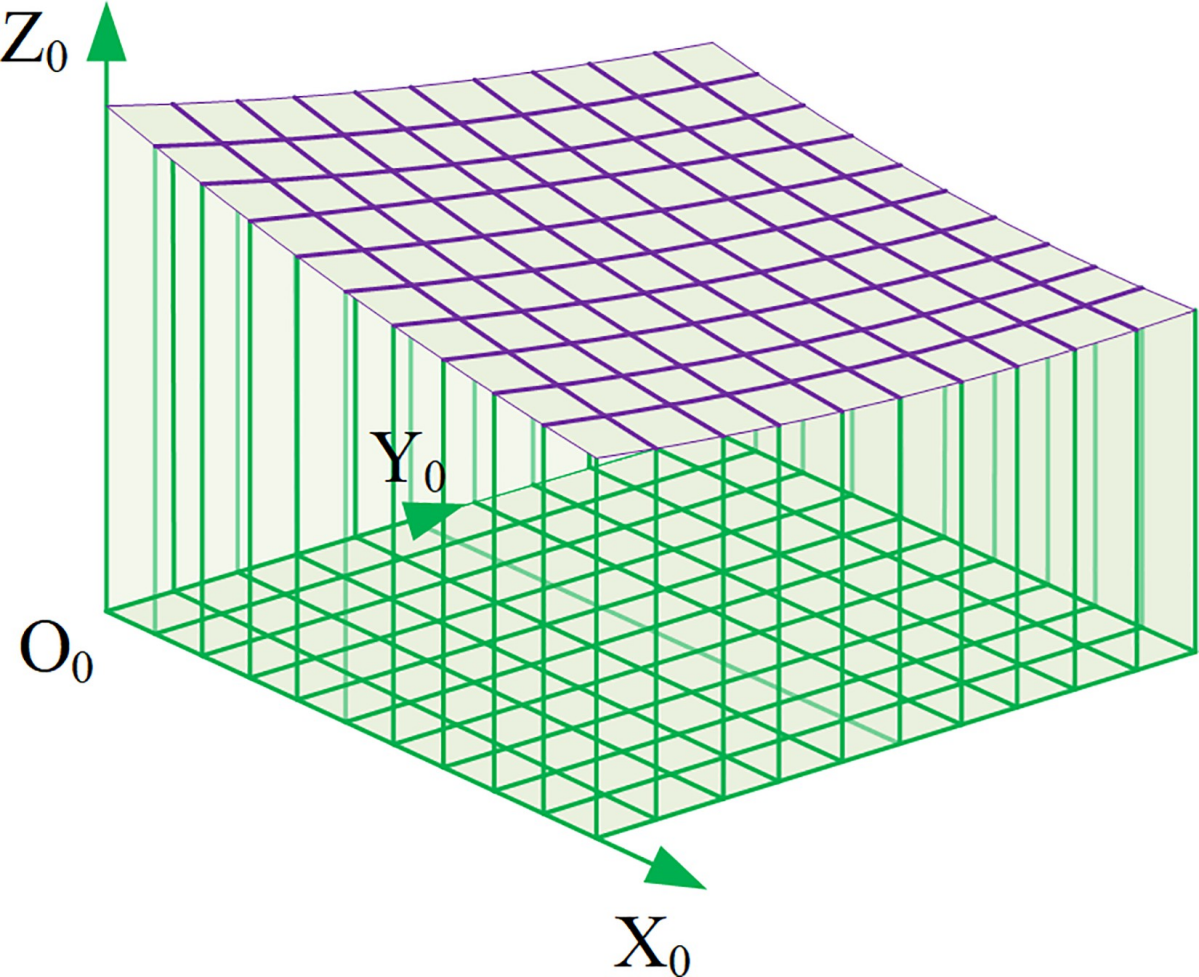
Z-map model.

The Z-map model is an effective technique that utilizes virtual points in three-dimensional space to describe three-dimensional objects. It can effectively capture details in three-dimensional space, thereby better obtaining the true structure of objects, as shown in Fig 6.

The most important part of Z-map is the description of Z-axis data, which is the main data required for final processing. By real-time detection of whether the Z-axis height has changed, it can determine whether the position point has changed. In this way, the detected 3D data is converted into binary images for post-processing. At first, the Z-map algorithm was an effective method for describing trajectories in three-dimensional space. It could project complex curves or surfaces onto the X-Y plane to achieve precise positioning of space. By subdividing the plane area into several grids, the height Z value of each grid node could be calculated and recorded for further research. According to Formula 1, the grid can be accurately divided, which can improve the accuracy of the simulation. The characteristics of this model are intuitive expression, adjustable grid density, simple structure, and resource saving.


$$\left\{ \begin{array}{l} x=x_{0}+i=Ax\\ y=y_{0}+j=4j \end{array}\right.$$ (8)

Therefore, in this article, the Z-map algorithm can be used to determine in real-time whether the roadway roof has been cut by the cutting head of the tunneling machine. By determining the left side of the contact position between the cutting head and the roadway roof, the spatial trajectory and three-dimensional surface during the cutting head’s movement process can be expressed. A series of discrete Point data are used to store the height of the roadway roof in the spatial position coordinates before cutting. Then, by comparing the relationship between the z-values of the point under the motion trajectory of the cutting head, it is determined whether the Point participates in cutting. If it participates in cutting, the Z-axis position data is updated in real-time. The final roof morphology is fitted by continuously updating the height data of discrete points during the cutting process.

### Tunnel contour simulation

According to the expression equation of the external contour equation in the above formula, combined with the position coordinate transformation between the cutting head coordinate system and the tunnel coordinate system, the external contour position of the cutting head under the corresponding feed rate and rotary table angle can be obtained.

When using Matlab for simulation of tunneling machine operation, taking the actual working conditions of the 122304 working face of Zhamei Special Preparation Coal Mine as the background, the EBZ160 tunneling machine was used. Based on the structural characteristics of the EBZ160 tunneling machine, the relevant parameters during the simulation process were precisely adjusted. The specific parameters are shown in Table 2.

**Table 2:** Simulation parameter setting of roadway roof.

Parameter code	Parameter value	Parameter code	Parameter value
*l*_*0*_ */mm*	0	*l*_*1*_ */mm*	3000
*l*_*2*_ */mm*	700	*l*_*3*_ */mm*	2470
*l*_*4*_ */mm*	1775	*h*_*1*_ */mm*	1000
*h*_*2*_ */mm*	519	*h*_*3*_ */mm*	87
*h*_*4*_ */mm*	120	*l*_*01*_ */mm*	400
*l*_*02*_ */mm*	600	*dl*_*3*_ */mm*	0
*θ*_*0*_ */°*	30	*θ*_*2*_ */°*	40
*r*_*1*_ */mm*	254	*n*_*1*_ */mm*	360
*n*_*2*_ */mm*	200	*β/°*	73
*α/°*	73	*ϖ*/(rad/s)	0.006

**Fig 7:**
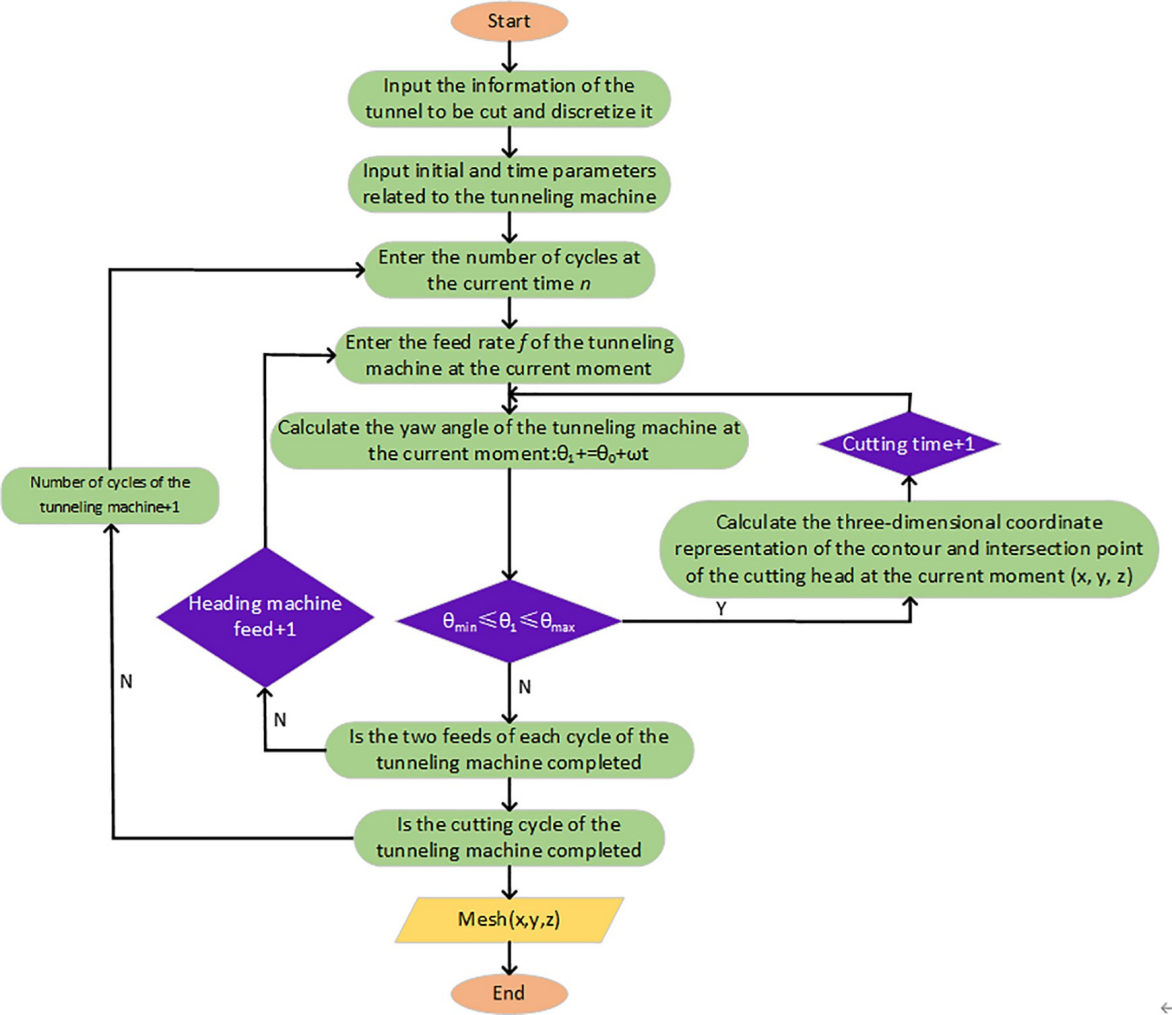
Flow chart of roadway morphology of Z-map algorithm.

By discretizing, obtain the coordinates of the corresponding discrete points under the contour curve. By recording the coordinates of key cutting points throughout the entire cutting process, subtracting the coordinates of discrete points on the outer contour of the cutting head from the original roadway roof coordinates, and performing surface fitting, the surface morphology of the roadway under the influence of the cutting head shape of the tunneling machine during the full section cutting process can be obtained. Using MATLAB software and Z-map algorithm for simulation, the simulation process is shown in Fig 7.

**Fig 8:**
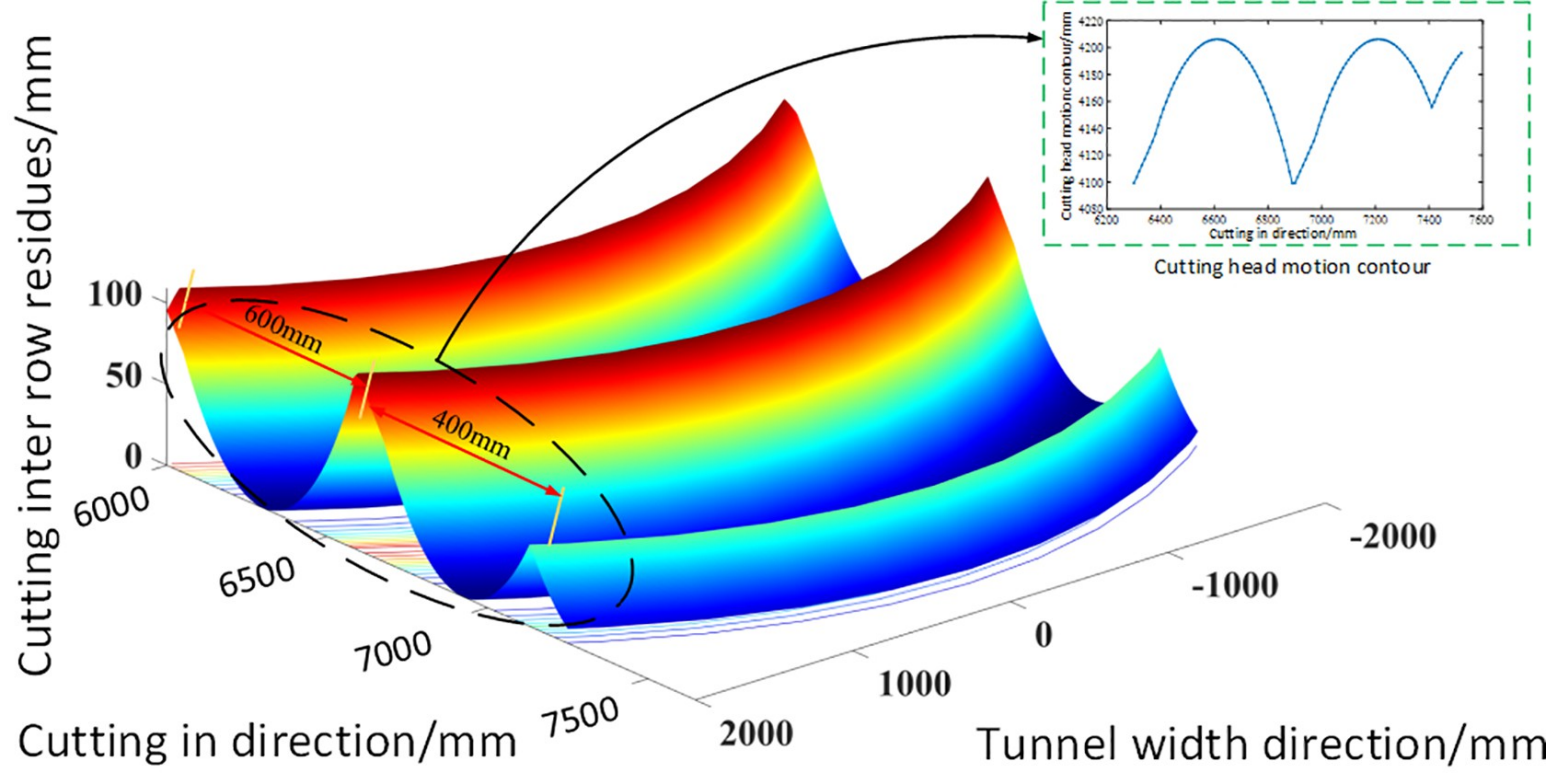
Simulation results of cutting inter row residues.

Finally, the simulation results of the morphology of the roadway roof after cutting are shown in Fig 8. In the figure, due to the feeding process of the cutting head, pits and ridges will be formed on the roof between adjacent feeds. At the same time, due to the lateral swinging effect of the cutting head, the residual peaks and ridges between the cutting lines show a clear trend of fan-shaped surfaces. The simulation results have well verified the expected results.

### Description of tunnel contour for variable cutting process under different tunnel widths

**Fig 9:**
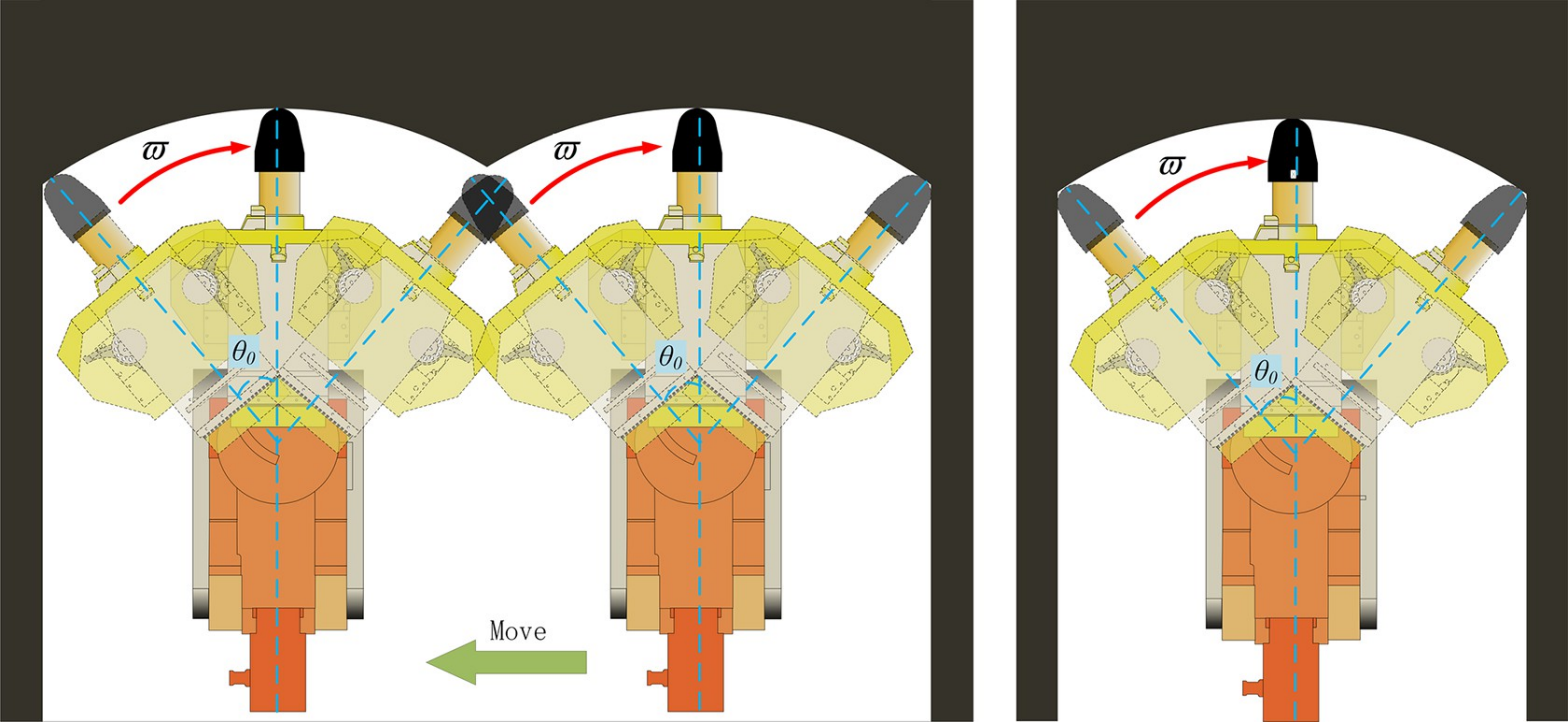
Working condition of wide and narrow roadway.

In the process of underground excavation, due to the complexity of tunnel working conditions, there are often different cutting processes. As shown in Fig 9, when the tunnel width is too wide and the tunneling machine cannot complete full section cutting with one feed, it is necessary to move horizontally in the tunnel and adjust the cutting position to modify the cutting section of the tunnel. Due to the limitations of the lateral movement of the cutting head of the tunneling machine, the cutting trajectory of the subsequent lateral movement will intersect with the circular arc motion trajectory of the previous lateral movement, resulting in a change in the residual shape of the roof after cutting under this working condition.

**Fig 10:**
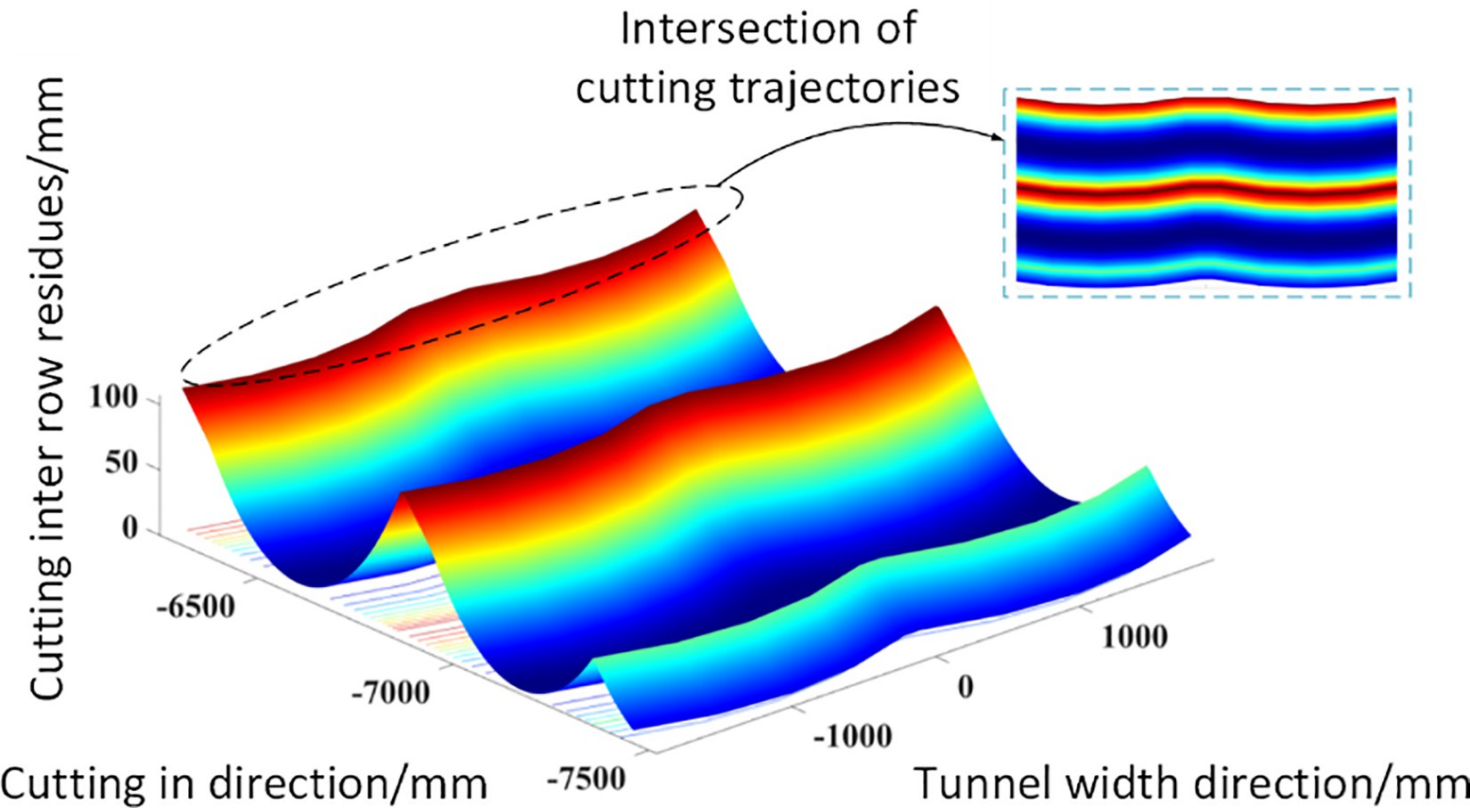
Simulation results of residual between cutting rows in wide tunnels.

For the morphology of the top plate of the excavation machine roadway under this working condition, simulation was continued using the Z-map algorithm to obtain the residual results between rows after the top plate was cut, as shown in Fig 10. It can be seen from the figure that under the two horizontal cutting conditions of the wide roadway, there are also peaks and ridges in the residual between rows after the top plate is cut. At the same time, due to the intersection of the two cutting trajectories, the residual peaks and ridges after cutting appear as wavy fan-shaped residues.

### Research on the topplate morphology under the vibration of cutting head’s lateral swing

Due to the complexity of the shape of the cutting teeth, the contact point between the cutting teeth and the roadway will change during the lateral cutting process of the cutting head. This makes the influence mechanism of the binding force and physical factors on the surface morphology more complex, especially in the cutting process of the broken roof. Due to the effect of vibration, the dynamic displacement of the cutting head will have a great impact on the surface morphology, directly affecting the flatness of the lateral roof. Therefore, it is particularly important to study the transverse shape model of the cutting roof under the dynamic response of the cutting system.

**Fig 11:**
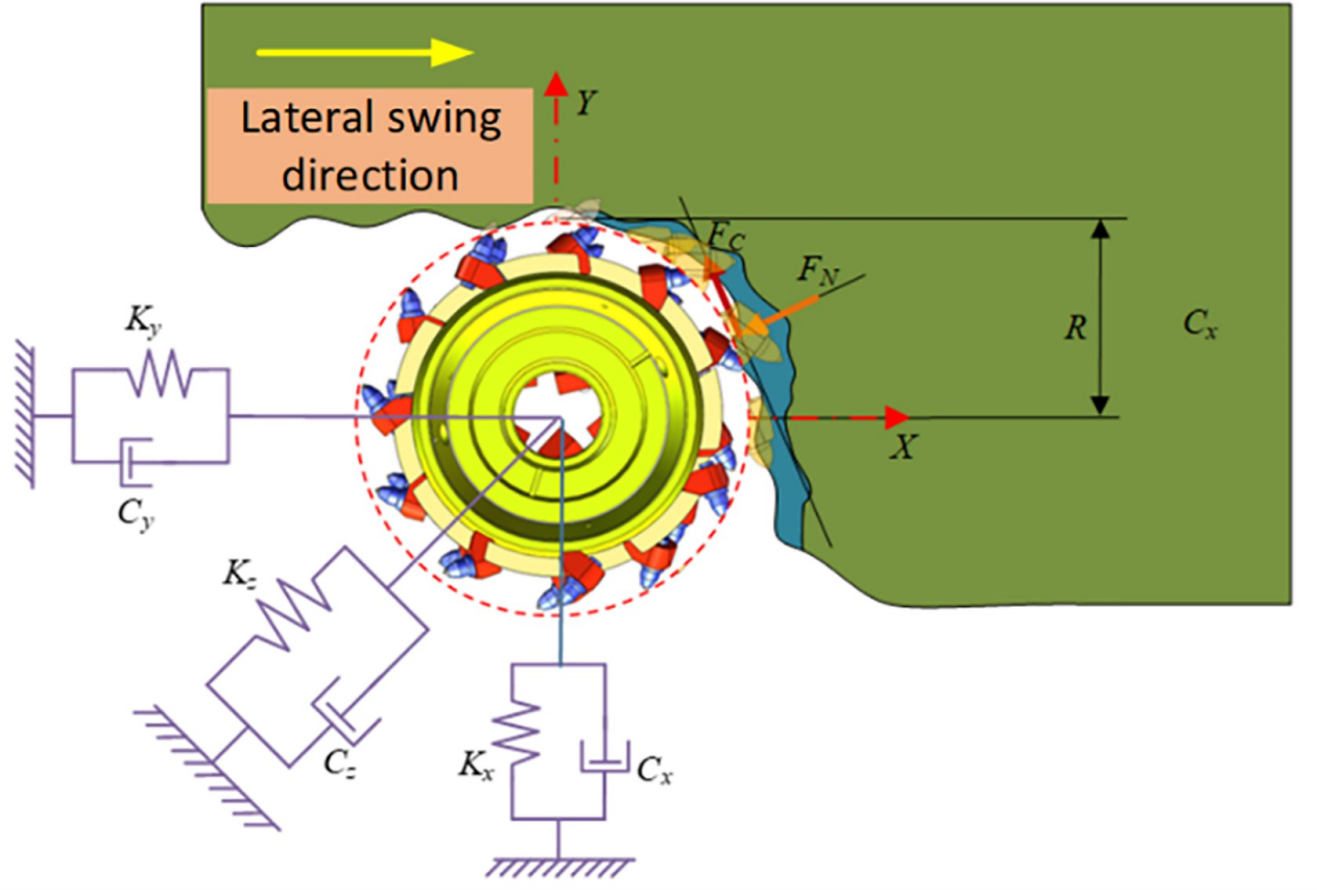
Dynamic model of cutting head system.

In the cutting system, vibration excitation can not only generate static cutting force, but also dynamic cutting force that changes over time. This dynamic cutting force is generated by the regenerative effect of the cutting process, as shown in Fig 11. The actual cutting thickness not only includes the nominal thickness, but also includes the regenerative cutting thickness caused by the dynamic displacement of the cutting teeth and coal wall induced by vibration, which causes changes in cutting thickness [17–19].

During the cutting process, the cutting teeth are subjected to the resistance of the coal and rock ahead of the cutting speed, the downward compression of the coal and rock during cutting is subjected to the reaction force of the coal and rock, and the cutting teeth are also subjected to the reaction force on both sides of the cutting groove. Through the analysis of the spring damping system in three directions, we can better understand the dynamic characteristics of the cutting process and better control the cutting effect:

$$M\ddot{s}(t)+C\dot{s}(t)+Ks(t)=F(h,t)$$ (9)

$$\begin{array}{l} M=\left[\begin{array}{ccc} m_{x} & & \\ & m_{y} & \\ & & m_{z} \end{array}\right],K=\left[\begin{array}{ccc} m_{x}\omega_{nx}^{2} & & \\ & m_{y}\omega_{ny}^{2} & \\ & & m_{z}\omega_{nz}^{2} \end{array}\right]\\ C=\left[\begin{array}{ccc} 2m_{x}\zeta_{x}\omega_{nx} & & \\ & 2m_{y}\zeta_{y}\omega_{ny} & \\ & & 2m_{z}\zeta_{z}\omega_{nz} \end{array}\right],s(t)=\left[\begin{array}{c} x(t)\\ y(t)\\ z(t) \end{array}\right] \end{array}$$ (10)

In the formula, *M*, *C*, and *K* are the modal mass matrix, damping matrix, and stiffness matrix of the cutting head, respectively; *m*_*x*_,*m*_*y*_,*m*_*z*_,*ζ*_*x*_,*ζ*_*y*_,*ζ*_*z*_,*ω*_*nx*_,*ω*_*ny*_,*ω*_*nz*_ are the modal masses, damping ratios, and natural frequencies in the x, y, and z directions respectively; *s(t)* represents the tool vibration displacement at the current moment; *F(h*,*t)* is the cutting load on the cutting head, which is related to the cutting thickness and time [20].

The dynamic cutting thickness *h*_*i*_*(t)* can be comprehensively represented by the theoretical cutting thickness *h*_*0*_, the dynamic displacement *s*_*i*_*(t-T)* of the previous cutting tooth, and the dynamic displacement *s*_*i*_*(t)* of the subsequent cutting tooth:

$$h_{i}(t)=h_{0}+[s_{i}(t-T)-s_{i}(t)]$$ (11)

At the same time, due to the influence of the distribution and form of the cutting teeth, as well as the vibration of the cutting head, the time delay of the cutting head in the coal and rock cutting process will vary with the changes in the cutting teeth participating in the cutting, the micro elements on the cutting teeth, and the cutting time of the cutting teeth participating in the cutting. Therefore, it is necessary to accurately determine the number and position of cutting teeth involved in cutting:

$$g(\varphi_{i}(t))=\{\begin{array}{l} 1\;\omega_{in}\leq \omega_{i}\leq \omega_{out}\\ 0\;other \end{array}$$ (12)

*g*(*φ*_*i*_(*t*)) is a function that determines whether the cutting teeth participate in cutting; where*ω*_*in*_ is the starting cutting position of the i-th cutting tooth and *ω*_*out*_ is the ending cutting position. For counterclockwise cutting, *ω*_m_ = 0, $\omega_{\mathrm{out}}={\mathrm{arcos}(1‐a}_{e}/R)$; for clockwise cutting, $\omega_{\mathrm{in}}={\mathrm{arcos}(1‐a}_{e}/R)$, *ω*_out_ = *π*; where a_e_ is the radial cutting depth and R is the cutting head radius.

Therefore, the cutting load at this time can be expressed as:

$$F(h,t)=F(g,h,t)=[\begin{array}{c} \sum_{i=1}^{N(h_{d})}g(\varphi_{i}(t))F_{X}(h,t)\\ \sum_{i=1}^{N(h_{d})}g(\varphi_{i}(t))F_{Y}(h,t)\\ \sum_{i=1}^{N(h_{d})}g(\varphi_{i}(t))F_{Z}(h,t) \end{array}]$$ (13)

For the cut vibration equation containing time-delay response mentioned above, the discrete interpolation method proposed by Xie Miao et al. [21] is used to solve the state space equation. The dynamic displacement *x(t)*, *y(t)*, and *z(t)* of the cutting head in the cutting system of the tunneling machine can be obtained.

In order to more accurately study the dynamic displacement changes of cutting caused by vibration, we must re-examine the proposed kinematic model. Due to the fact that the dynamic displacement of the cutting head is described in the cutting head coordinate system, and in the tunnel coordinate system, the homogeneous transformation matrix describing the cutting head can be obtained through _4_*T*. Therefore, coordinate transformation is carried out on the key nodes *O*_*4*_, *O*_*j*_, *C*_*1*_, *C*_*2*_, *C*_*3*_ (j = 1, 2, 3, 4) in the cutting head coordinate system. Use $p_{d}=[\begin{array}{cccc} x(t) & y(y) & z(t) & 1 \end{array}]$ to represent the dynamic displacement coordinates of the key position morphology of the cutting head in the cutting head coordinate system. Based on the geometric motion and vibration induced effects mentioned earlier, we can express the relationship between the key points of the cutting head and its coordinate system as follows:

$$\begin{array}{l} {\;}^{4}{\tilde{p}}_{O4}={\;}^{4}p_{O4}+p_{d}\\ {\;}^{4}{\tilde{p}}_{Oj}={\;}^{4}p_{Oj}+p_{d}\\ {\;}^{4}{\tilde{p}}_{C1}={\;}^{4}p_{C1}+p_{d}\\ {\;}^{4}{\tilde{p}}_{C2}={\;}^{4}p_{C2}+p_{d}\\ {\;}^{4}{\tilde{p}}_{C3}={\;}^{4}p_{C3}+p_{d} \end{array}$$ (14)

The coordinate expression of the cutting head under vibration induction in the tunnel coordinate system can be obtained by the following formula:

$$p{=}_{4}T^{4}p={\left[x_{p}\;y_{p}\;z_{p}\;1\right]}^{T}$$ (15)

## Research on roof roughness based on fractal theory

### Basic fractal theory

**Fig 12:**
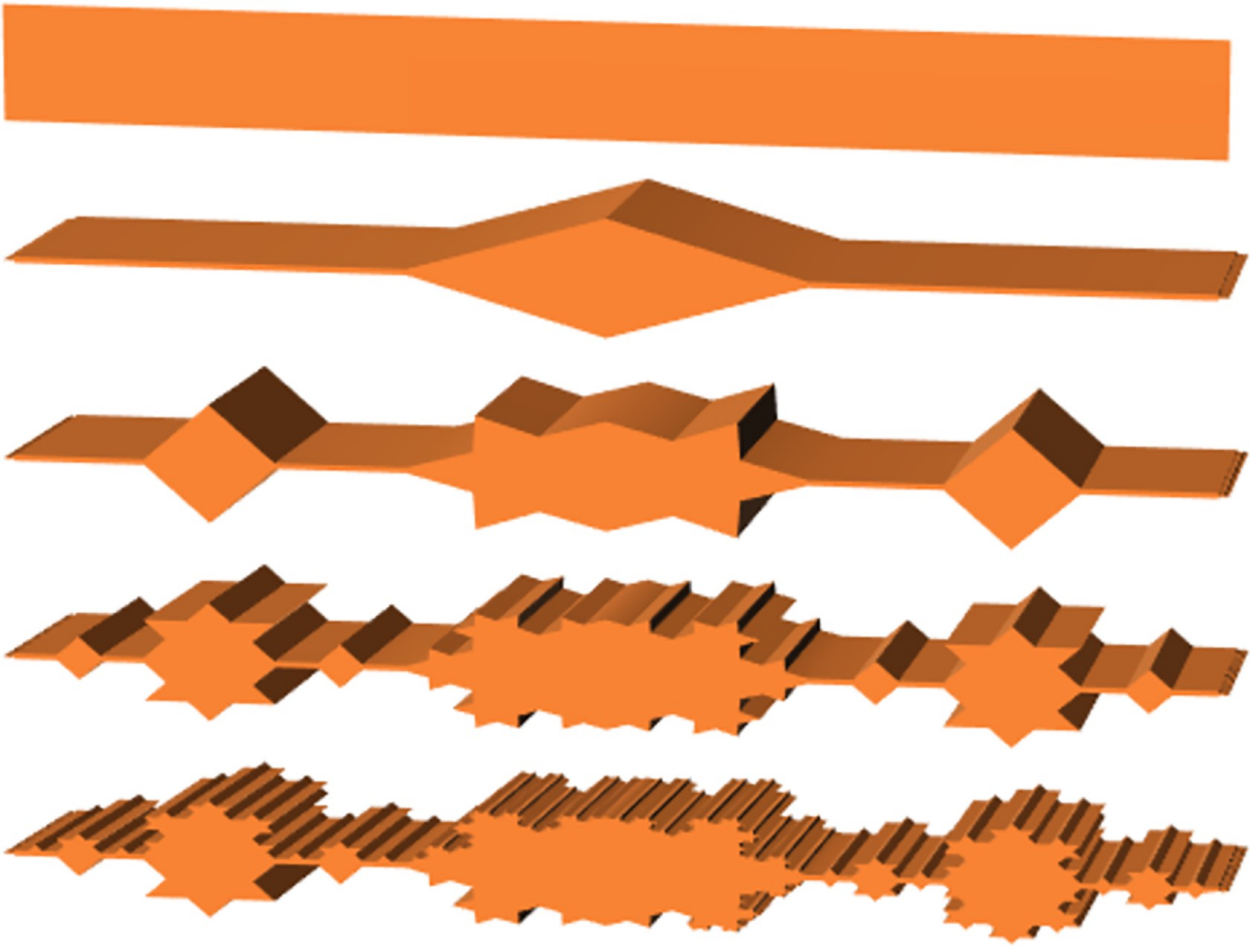
The ternary Koch curve of fractal theory.

In practical engineering applications, fractals usually refer to fractals that have self similarity in statistical sense, that is, they only have self similarity within a certain range. Fractal technology can be used to simulate the structure of underground spaces, in order to better meet the growing needs of mining, installation, and maintenance, which cannot be objectively described by traditional geometry. Therefore, using fractal geometry methods to quantitatively characterize the surface morphology of coal and rock has obvious advantages, as shown in the fractal theory diagram in Fig 12.

In practical engineering applications, fractals usually refer to fractals that have self-similarity in a statistical sense, that is, they only have self-similarity within a certain range. Fractal technology can be used to simulate the structure of underground spaces, in order to better meet the growing demand for excavation, installation, and maintenance, which traditional geometry cannot objectively describe. Therefore, using fractal geometry methods to quantitatively characterize the surface morphology of coal and rock has obvious advantages.

This article uses the W-M function method improved by Yan et al. to describe the characteristics of the rough morphology of the cut roof, as shown in Eq (16).


$$\begin{array}{c} z(x,y)=L{\left(\frac{G}{L}\right)}^{\left(D-2\right)}{\left(\frac{\ln\gamma }{M}\right)}^{1/2}\sum_{m=1}^{M}\sum_{n=0}^{n_{\max}}\gamma^{(D-3)n}\{\cos\phi_{m,n}-\\ \;\cos[\frac{2\pi \gamma^{n}{\left(x^{2}+y^{2}\right)}^{1/2}}{L}\cos(\arctan(\frac{y}{x})-\frac{\pi m}{M})+\phi_{m,n}]\} \end{array}$$ (16)

Among them, z(x,y) represents the roughness of the surface layer of the roadway, which can be used to measure the maximum height difference between wave peaks and valleys. While D is the fractal dimension on the three-dimensional roughness surface. For three-dimensional fractal surfaces, its value is usually between 2–3, G is the characteristic size coefficient describing the height change of the surface layer, γ can be used to measure the frequency density, usually γ is taken as 1.5, and Ls is the shortest truncation distance describing the rough surface, n is an indicator used to describe the specific frequency of the surface layer, x and y are coordinate parameters of the spatial position of the roof, and:

$$n_{\max}=\mathrm{int}[\frac{\log(L/L_{s})}{\log\gamma }]$$ (17)

*L* is the sampling length of the rough surface, *M* is the overlap number of surface wrinkles,*φ*_*m*_, *n* is the random phase, and its value range is [0,2π]. According to Eq 16, the height of rough surface contours is mainly influenced by fractal dimension and feature scale coefficients.

### Determination of key fractal parameters

**Fig 13:**
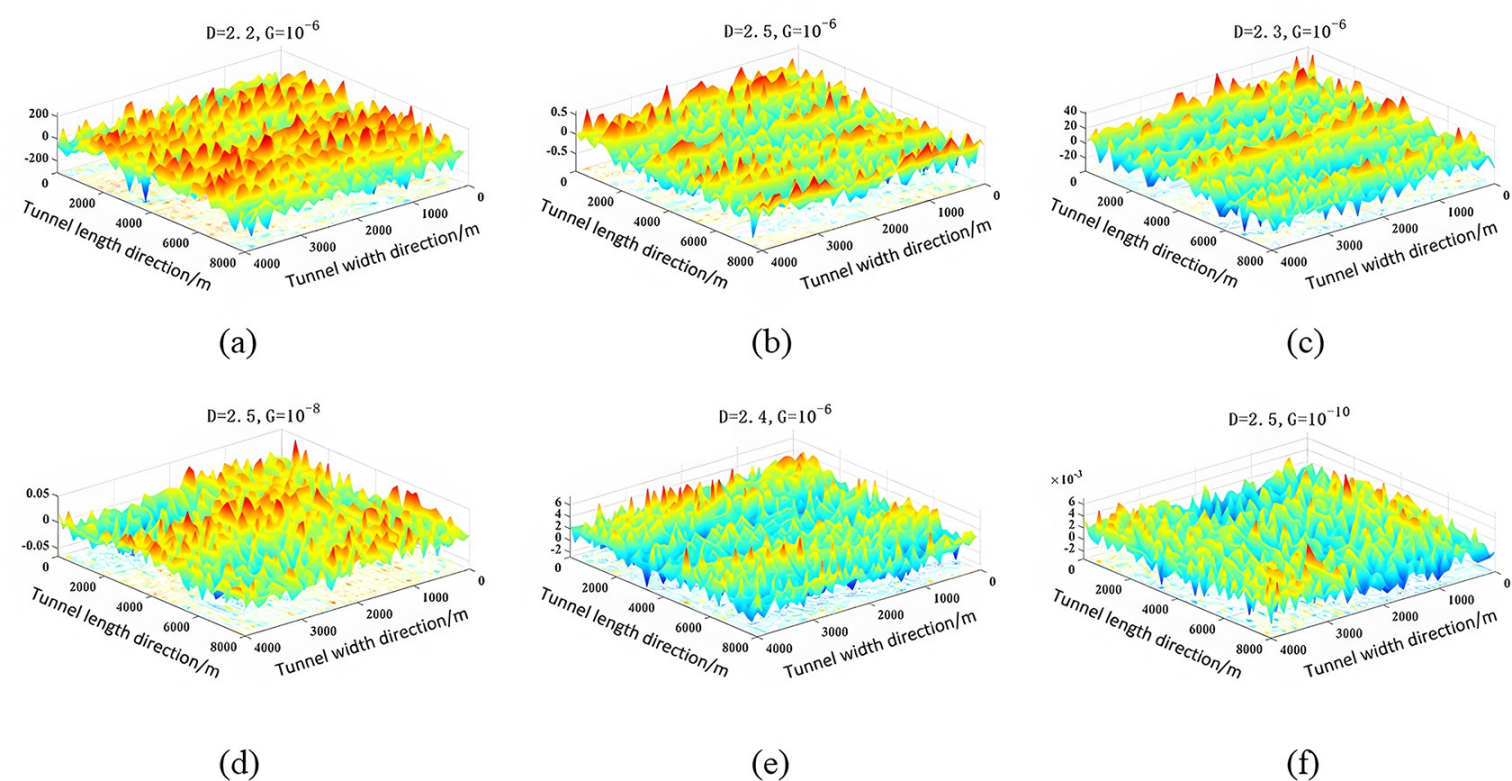
3D fractal surface morphology.

Due to the brittleness of the coal and rock in the roadway roof, the roof after cutting is in a state of falling and crushing, resulting in a high surface roughness of the cut roof. In order to consider the influence of the above factors, L = 256, Ls = 1, M = 5 are selected. Through the above formula, the changes in surface roughness of the roof under different fractal dimensions D and characteristic scale coefficients G can be obtained, as shown in Fig 13.

It can be clearly seen from the above figure that the fractal dimension D and feature scale coefficient G significantly affect the fineness of rough surface morphology. Through the modified isotropic three-dimensional fractal rough surface model, it was found that with the increase of fractal dimension and feature scale coefficient, the scale range of the simulated rough surface gradually decreases. At this point, the surface of the roadway exhibits finer microscopic morphological features, and the roof is smoother at the macro scale with lower roughness. On the contrary, as the fractal dimension decreases and the feature scale coefficient increases, the roughness scale of the simulated rough surface significantly increases, and the roughness of the simulated roadway roof is higher at this time.

**Fig 14:**
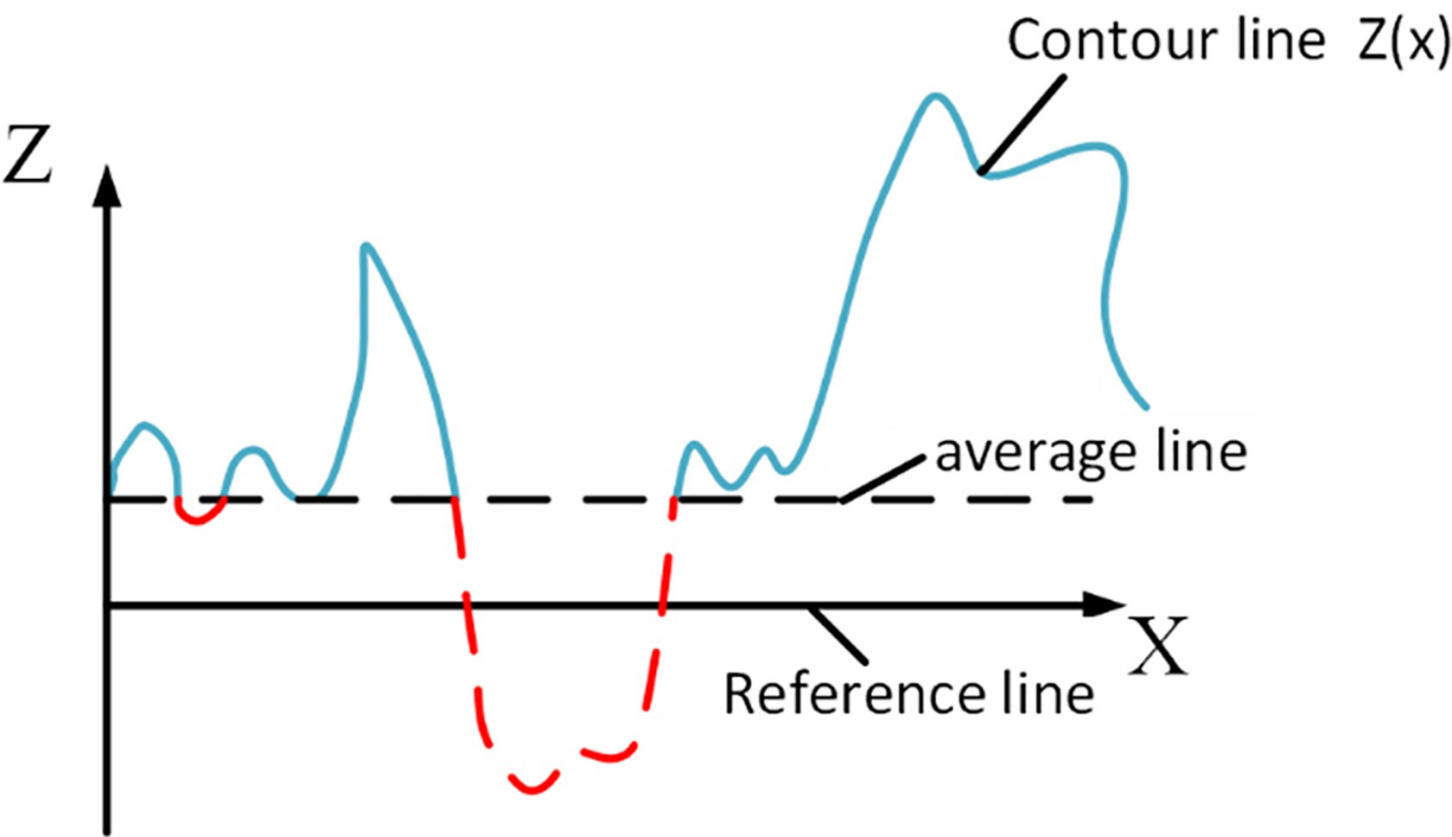
Schematic diagram of surface contour line.

In order to more clearly represent the relationship between the roughness performance of fractal theory and related factors, traditional methods often measure the size of surface roughness by introducing the root mean square value of the relative deviation between the standard contour line and the average size line when conducting research on the roughness of two-dimensional fractal theory, as shown in Fig 14.

By introducing*σ(D*, *G)*, the roughness of simulated rough surfaces can be intuitively expressed as a function of fractal scale D and feature scale coefficient G:

$$\sigma (D,G)=\sqrt{\frac{G^{2(D-1)}}{2\ln\gamma }\frac{1}{4-2D}L^{4-2D}}$$ (18)

Wu Bing [22] extended the above method to the field of three-dimensional fractal. Since in three-dimensional fractal theory, the fractal dimension D and two-dimensional fractal dimension Ds satisfy the expression rule of D = Ds+1, the root mean square error *σ’(D*, *G)* in the height direction of three-dimensional rough surfaces can be expressed as:

$$\sigma^{\prime }(D,G)=\sqrt{\frac{G^{2(D-2)}}{2\ln\gamma }\frac{1}{6-2D}L^{6-2D}}$$ (19)

From the above Formula 18, it can be seen that changing the different fractal dimensions D and the characteristic scale coefficient G of the roof coal and rock can more intuitively depict the trend of changes in the surface roughness of the roadway through the curve, thus better understanding the variation law of the roadway roughness.

**Fig 15:**
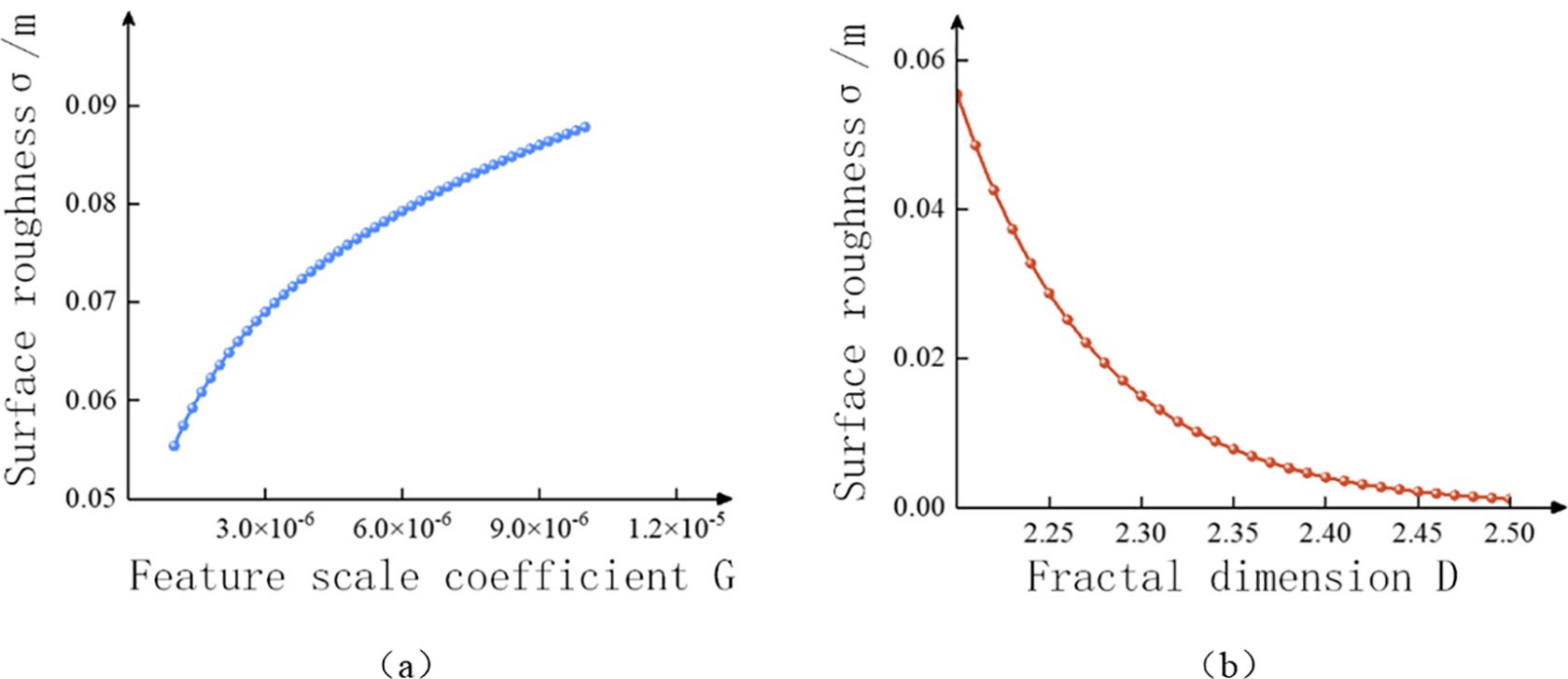
Roughness characteristics under the influence of fractal parameters.

As shown in Fig 15, when the feature scale coefficient G is constant and the fractal dimension D decreases, the standard deviation*σ’(D*, *G)* of surface height increases accordingly. As the fractal dimension increases, the roughness degree on the rough surface will gradually decrease, this change relationship is nonlinear, and the trend of change will also gradually slow down. Under a given fractal dimension, as the feature scale relationship increases, the surface roughness will also improve, and the trend of change will gradually slow down as the fractal dimension increases.

Therefore, when simulating the morphology of the rough roof of the initial excavation tunnel, the distribution law of the simulated rough roof generated by fractal theory is studied, and the feature scale coefficient G and fractal dimension D are adjusted based on the roughness characteristics under the influence of classification parameters, so that the variation and distribution of the generated rough roof in the height direction are close to the actual roof law of the tunnel surveyed on site.

**Fig 16:**
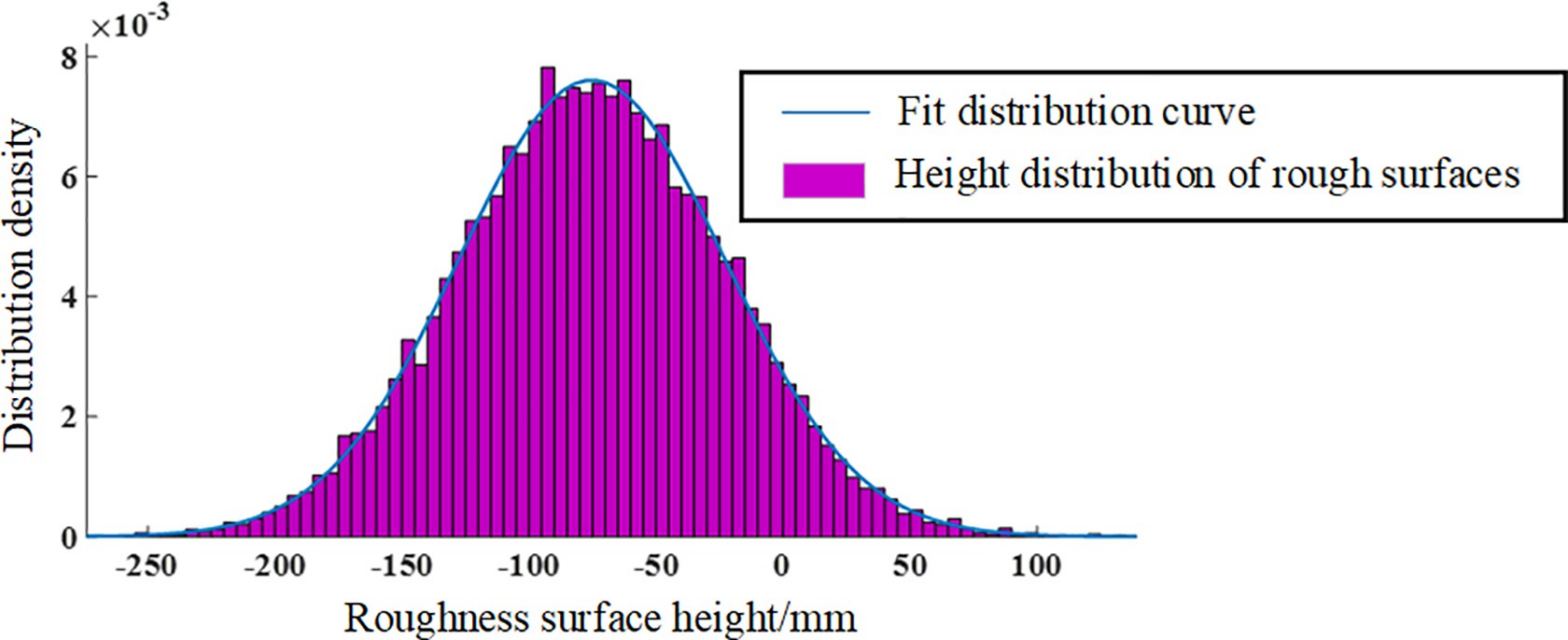
Distribution pattern of rough planes with D = 2.2 and G = 10–6.

Select the rough surface generated when D = 2.2 and G = 10–6 to explore the distribution pattern of the generated rough surface. The results are shown in Fig 16. It can be clearly seen from the figure that the rough roof generated by fractal theory shows a normal distribution trend, with the highest probability distribution density at the height of the rough surface near -100~-50m. At the same time, the range of rough surface roughness height is -250~200mm, and the maximum height difference between the peak and valley is 450mm. Through on-site investigation, we found that the maximum unevenness of the roadway roof reaches -300~300mm, which means the maximum height difference between the peak ridge and the concave valley is 600mm. Therefore, at this point, the surface roughness of the simulated roadway roof is relatively low, and it is necessary to appropriately reduce the fractal dimension and increase the characteristic scale coefficient to adjust the simulated roof.

### Establishment of roof model under multi factor coupling

**Fig 17:**
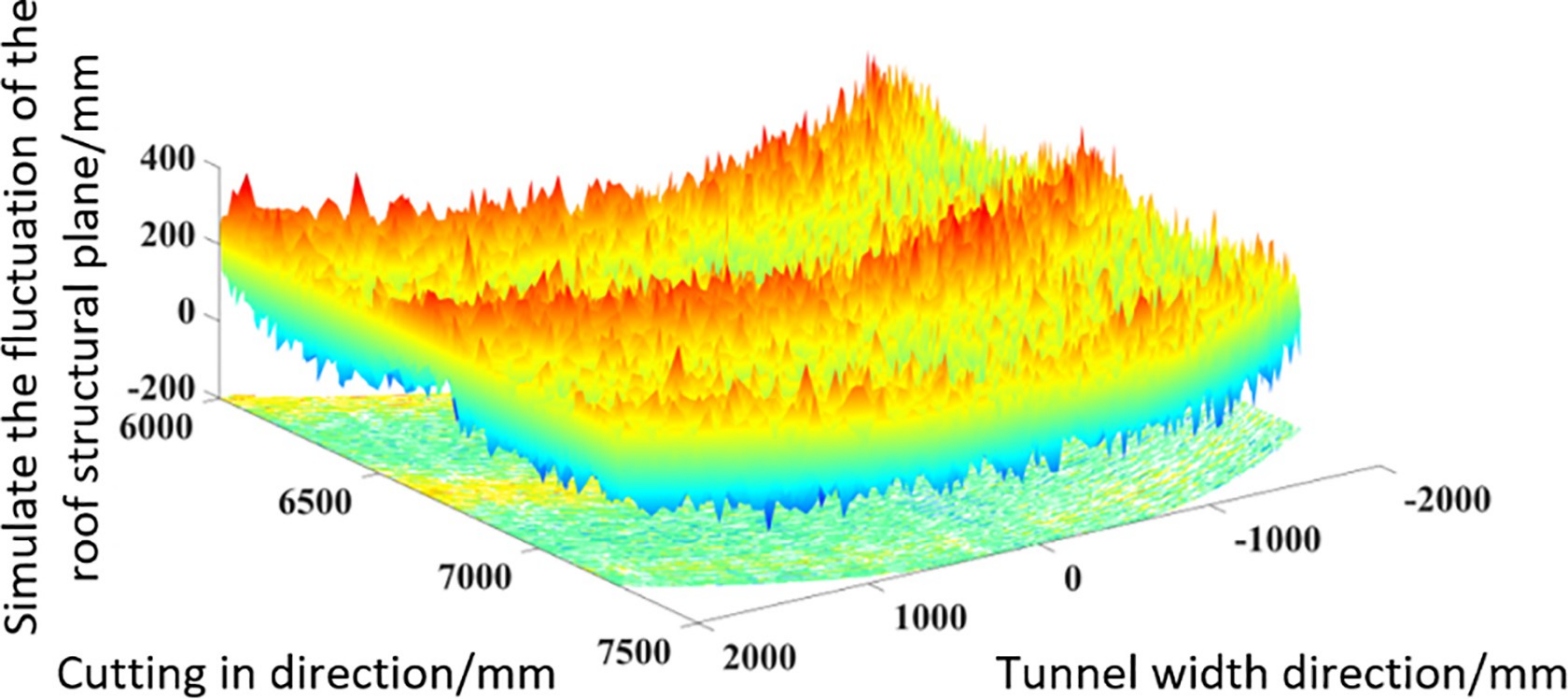
Simulated coal-rock rough surface 3D surface.

By combining the description methods of the three roofs morphology mentioned above, and overlaying the cut roof morphology characteristics under the influence of three factors, a quantitative evaluation method for the cut roof morphology characteristics can be obtained considering the cutting process, cutting vibration, and roof coal and rock collapse conditions. When simulating the actual roof of a roadway, we need to pre determine the characteristics of the surrounding rock on the roadway surface and the undulating characteristics of the rough surface after cutting. Based on the range value of the roadway surface morphology and the comprehensive expression of roughness, we need to determine the characteristic scale coefficient G and fractal dimension D of fractal theory. Finally, the roof morphology of the tunnel obtained from fractal theory is overlaid with the roof morphology under the influence of the cutting head considering cutting vibration obtained through the Z-map algorithm. By comprehensively calculating the data of the simulated tunnel roof using MATLAB, the quantified rough surface morphology of the coal and rock can be obtained, as shown in Fig 17.

Observing from the above figure, it is found that the three-dimensional surface of the rough roof obtained by combining the above three morphology description methods exhibits micro protrusions and depressions due to the collapse of coal and rock and the vibration induction of the cutting head at a small scale. However, due to the influence of cutting technology, it exhibits a state of excluding peaks, ridges, and valleys at a large scale.

## Experimental validation and data mapping

### Experimental verification

A 1:3 similarity experimental model of EBZ160 tunneling machine was used to build a cutting head coal rock system cutting experimental platform, and a comparative experiment was conducted on the cutting morphology. The motor power of the experimental platform was 7.5kW, the outer diameter of the cutting head was 350mm and 43 cutting teeth were installed, the cutting head body and tooth seat were made of 45 # steel. The specific parameters are shown in Table 3.

**Table 3:** Comparison of parameters between similar test bench and actual working conditions.

parameter	prototype	test bench model
*outer diameter of cutting head /mm*	D	*D/3*
*intercept spacing /mm*	L_jx_	L_jx_/3
*cutting tooth width /mm*	B	*B/3*
*cutting tooth height /mm*	h_c_	h_c_/3
*drilling rate /m*.*s*^*-1*^	v_s_	v_s_
*cutting head speed /r*.*min*^*-1*^	n	*3n*
*cutting force of the cutting head /KN95*	F	F/9
*coal rock density /kg*.*m*^*3*^	ρ	ρ
*compressive strength /MPa*	σ	σ

**Fig 18:**
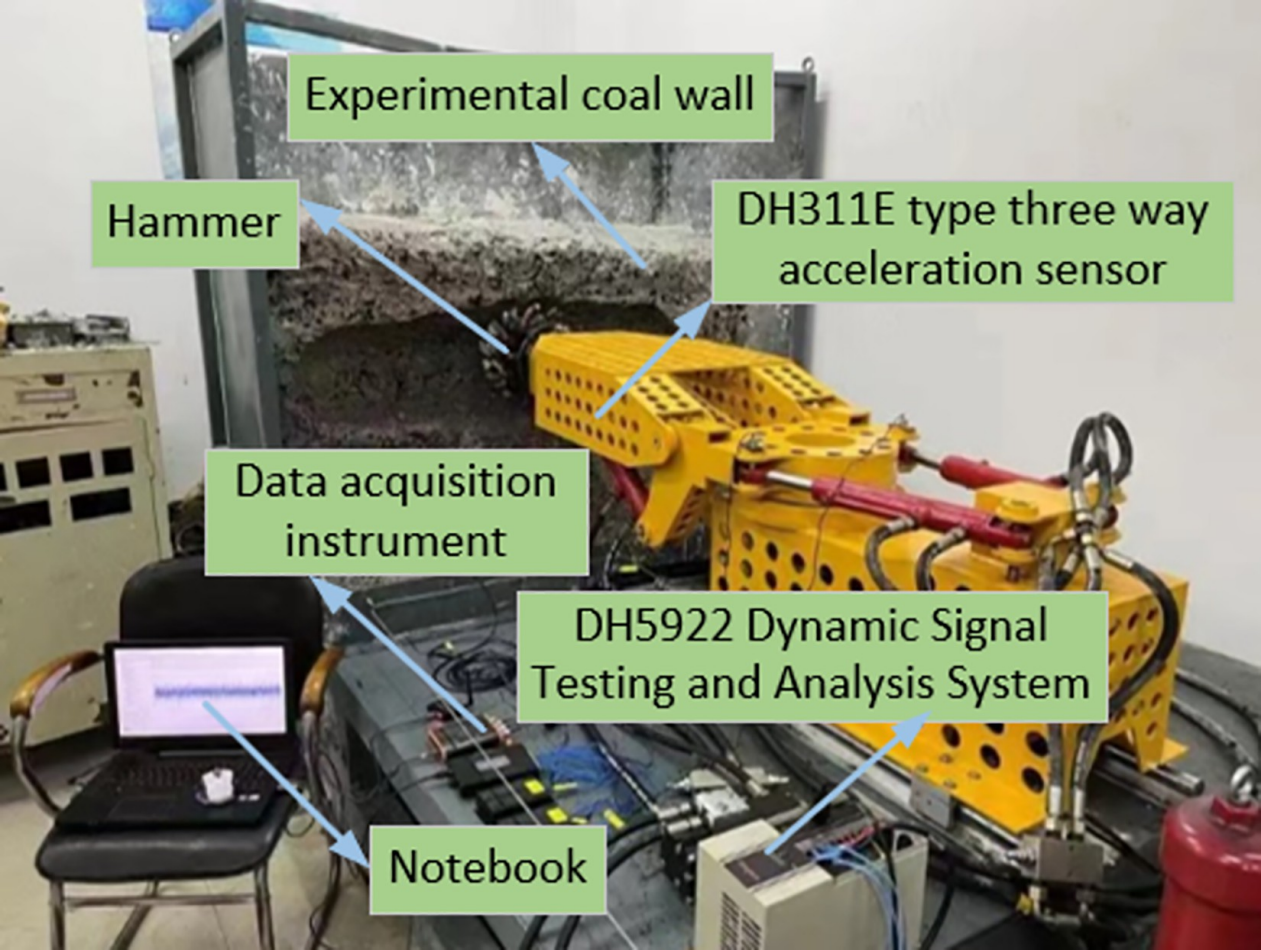
Coal and rock cutting similarity experimental platform.

During the testing process, in order to obtain vibration signals during the cutting process, Donghua DH5922 dynamic signal detection system was selected for collection. The DH311E three-dimensional acceleration sensor was selected and fixed on the tested unit. The experimental platform is shown in Fig 18.

**Fig 19:**
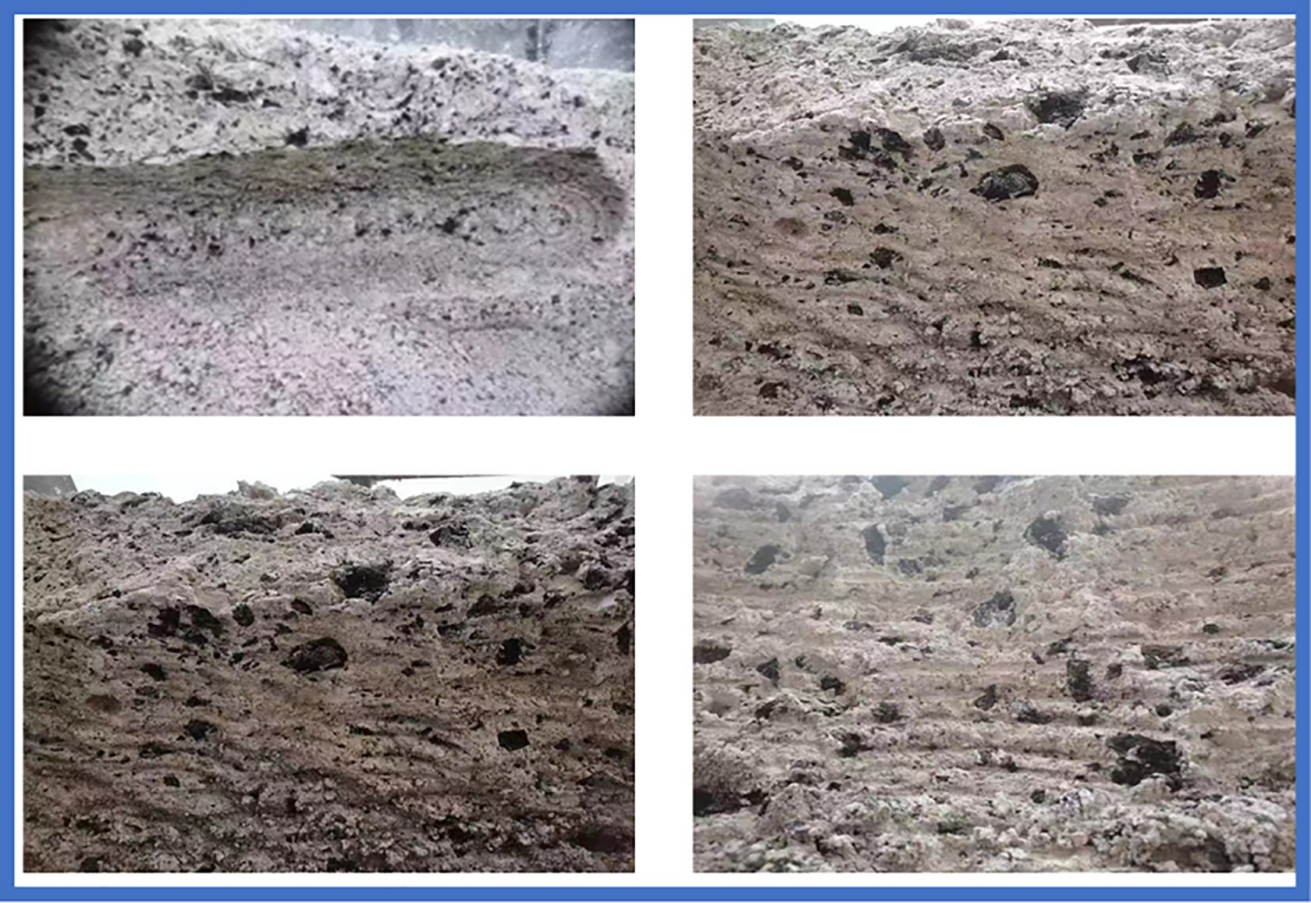
Surface of coal and rock after cutting.

In the experiment, when the power button is turned on, the force hammer starts cutting the experimental coal wall from left to right in sequence. The speed change of the force hammer is detected through a three-way acceleration sensor, and the acceleration information is transmitted to the dynamic signal testing and analysis system through a data acquisition instrument. After analysis by the dynamic signal detection and analysis system, the clear experimental results are displayed on a laptop for researchers to analyze and verify. The surface morphology of the coal rock after cutting was obtained through cutting similarity experiments. The surface morphology of the coal rock after cutting is shown in Fig 19.

At the same time, based on real-time research and sampling of coal mine tunnels, the maximum unevenness of the tunnel roof was obtained: -300~300mm, and the maximum peak ridge distance was obtained as 600mm. Combined with the rough coal rock image of the tunnel roof obtained from coal rock similarity experiments, the actual surface contour of the tunnel was constructed.

### Gray processing of roadway roof

Due to the fact that the photos taken by the camera are composed of three primary colors: red, green, and blue, which are represented as a three channel pixel matrix in MATLAB, the changes in the depth direction of the roadway roof cannot be directly represented through this three-dimensional array. However, grayscale images can well reflect the brightness difference of the displayed pixels in the photos. For the roof images obtained from underground exposure, the concave surface area may appear darker in the image due to less reflection of the shooting light, while the convex surface area may appear brighter in the image due to stronger camera exposure and reflected light intensity. Meanwhile, in MATLAB, grayscale images can be represented as a two-dimensional matrix with a value range between [0255]. Therefore, if normalization can be performed based on the average grayscale value of the image, and combined with the maximum peak ridge spacing obtained from actual research, a three-dimensional roof surface image based on actual roof images can be obtained.

**Fig 20:**
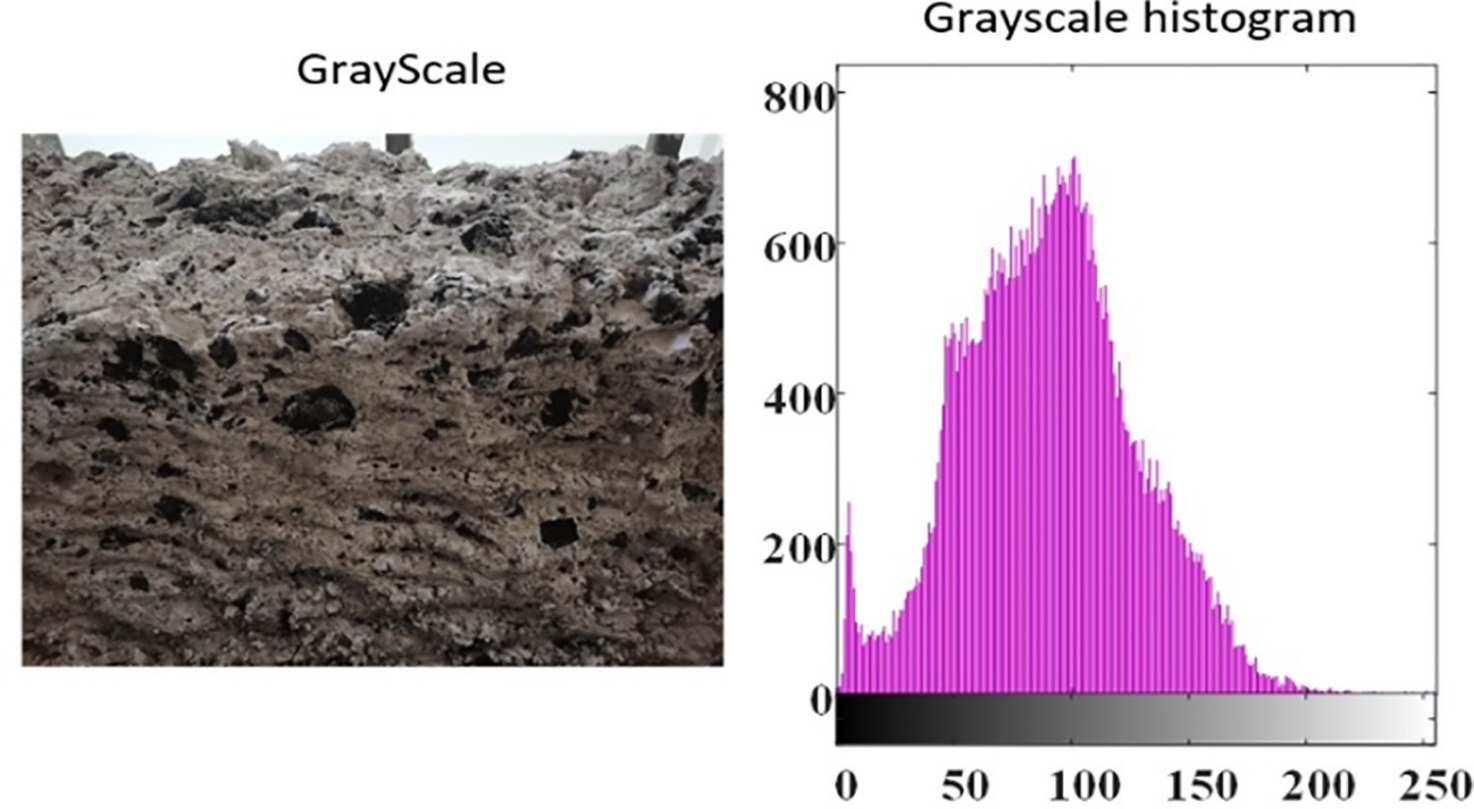
Gray scale and histogram of roadway roof.

By grayscale processing the image, the grayscale image and grayscale histogram of the roadway roof are obtained, as shown in Fig 20. From the figure, it can be seen that the grayscale histogram of the roof also exhibits a certain normal distribution characteristic, that is, the overall probability density of the rough peak and concave valley bottom of the roof is small, and the distribution frequency of the roof located in the middle layer is higher.

**Fig 21:**
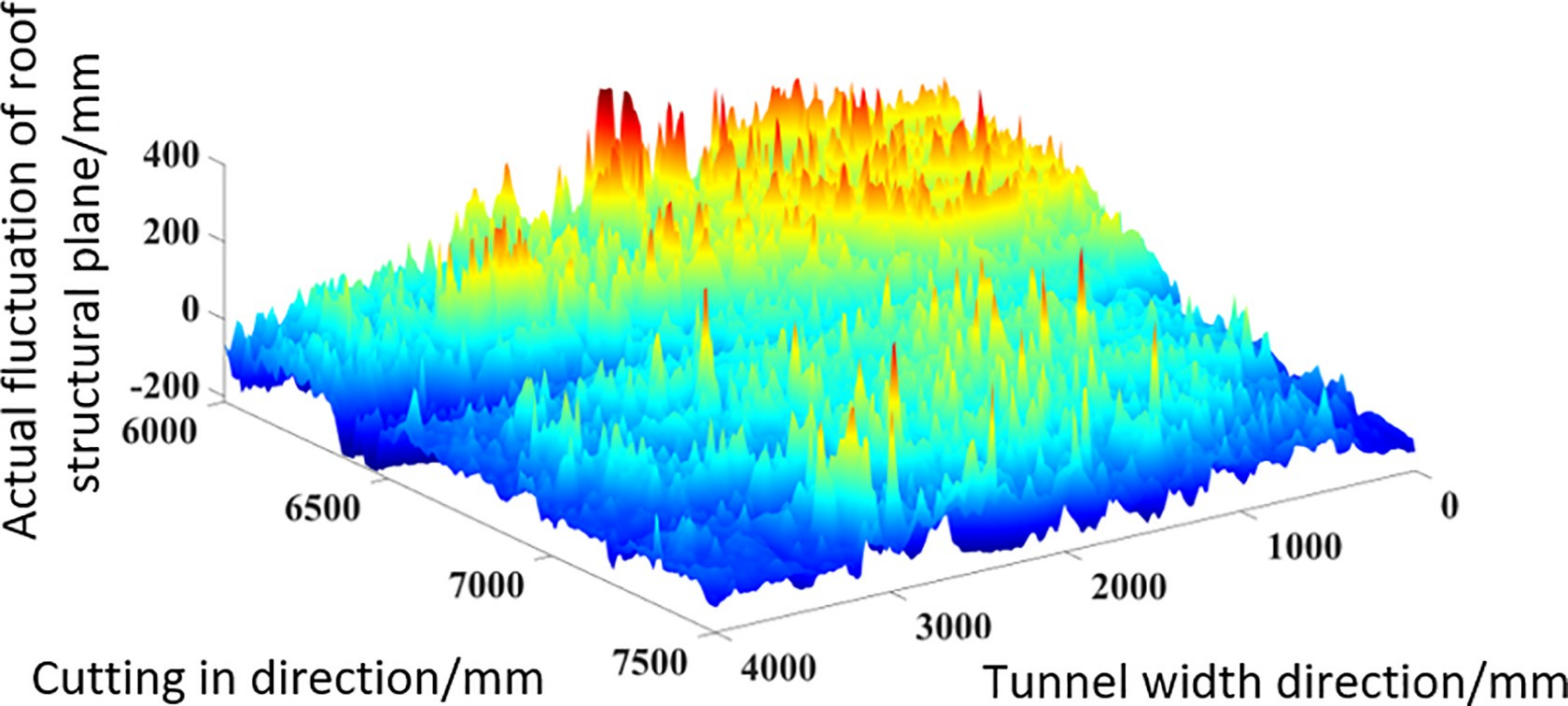
Real coal-rock rough surface 3D surface.

After grayscale processing of the image, the brightness of the image is normalized based on the grayscale value. Based on the peak valley range of 600mm, the maximum height of the peak ridge is 300mm, and the maximum depth of the valley bottom is -300mm, the image brightness is mapped to the range of [–300,300]. After completing the above data processing using MATLAB, the rough surface height fluctuation values corresponding to each pixel point in the image can be obtained. Finally, the three-dimensional surface diagram of the actual roof structural surface undulation was also completed using MATLAB, as shown in Fig 21.

As shown in Fig 21, the change process of surface color from blue to red in the figure reflects the transition process of image grayscale values from low to high, and also represents the roughness of the surface from low to high. From the actual three-dimensional surface graph, it can be found that in addition to small-scale rough peaks appearing locally, there are also obvious concave and convex peaks and valleys on the rough surface over a large scale range. There is significant similarity in size distribution and morphology between the rough surface and the simulated roof obtained through quantitative methods.

## Study on the effectiveness evaluation of simulated roof quantification methods

### Evaluation method for surface characteristics of roof

In the process of quantitative research on the surface morphology of rough roof slabs in tunnels, in order to make the structural surface undulations of the quantified roof slabs closer to the actual roof slabs, in addition to the maximum peak, valley, and maximum height differences considered in the previous section. It is also possible to compare the quantified structural surface feature parameters with the actual sampled tunnel roof parameters through statistical analysis, and evaluate the effectiveness of the quantification method. The commonly used evaluation values are marked with the following four types:

1. Maximum range of rough surface peaks and valleys
  Usually, range R is used to represent the distance difference between the maximum and minimum values of data, and is typically used to describe the size of the data range. This article uses the maximum difference between the peak ridge height *Z*_max_ and valley bottom *Z*_min_ on the coal rock after cutting as the maximum range *R*_max_ of the peak valley to evaluate the fitting effect of the overall roadway roughness.
$$R_{\max}=Z_{\max}-Z_{\min}$$ (20)
2. Standard deviation of rough surface height
  The standard deviation or mean square deviation *σ*_*z*_ is usually defined as the square root of the sum of squares of the sampled data deviating from the mean, usually represented by the letter *σ*, reflecting the degree of dispersion between individuals in the sampled data. This article uses the square root *μ* of the average sum of squared distances between the heights of each point on a rough surface and the mean surface *σ*_*z*_ to reflect the dispersion and volatility of the overall rough surface.
$$\sigma_{z}=\sqrt{\frac{\sum_{i=1}^{n}{\left(x_{i}-\mu \right)}^{2}}{n}},\mu =\frac{1}{n}(x_{i}+x_{2}+\cdots +x_{n})$$ (21)
3. Roughness surface wave peak skewness coefficient
  The skewness coefficient S can be expressed as the difference between the arithmetic mean and mode measured in standard deviation, or as the ratio of the third central moment to the third power of the standard deviation. It is commonly used to describe the distribution shape characteristics of a normal distribution. It reflects the degree of deviation from the average value of the data. If the skewness coefficient is greater than zero, it indicates that the data shows a positive bias trend, with the mean being higher than the median. If the skewness coefficient is less than zero, it indicates a negative bias trend in the data, with a median higher than the mean. In this article, the skewness coefficient reflects the degree of symmetry deviation in the distribution of rough surfaces by the distance between the mean surface and the median surface of rough structural surface fluctuations. When S = 0, the distribution is normal, when S<0, the distribution is left skewed (negative skewed), and when S>0, the distribution is right skewed (positive skewed).
$$S=\frac{\frac{1}{n}\sum_{i=1}^{n}{\left(x_{i}-\mu \right)}^{3}}{{\left(\frac{1}{n}\sum_{i=1}^{n}{\left(x_{i}-\mu \right)}^{2}\right)}^{\frac{3}{2}}}$$ (22)
4. Peaks and kurtosis coefficient of rough surface waves
  The kurtosis coefficient K, as an important reference for measuring the probability density distribution curve of a specific data distribution, can measure the characteristics of the curve by the sum of the fourth order center moment and the square of variance, thus better revealing the characteristics of the curve. In this article, the kurtosis coefficient is used to describe the peak height and aggregation degree of rough peaks. The higher the peak, the higher the deviation of low-frequency data from the average value. Their influence increases the variance. Usually, if the calculated kurtosis coefficient is greater than 3, it indicates that the data distribution density function is steeper than the normal distribution, and there is a certain range of deformation on the roof surface. However, if the kurtosis coefficient is less than 3, it indicates that compared to the normal distribution, the distribution density function curve of the roof surface changes more smoothly and the roof performance is smoother.
$$K=\frac{\frac{1}{n}\sum_{i=1}^{n}{\left(x_{i}-\mu \right)}^{4}}{{\left(\frac{1}{n}\sum_{i=1}^{n}{\left(x_{i}-\mu \right)}^{2}\right)}^{2}}$$ (23)

### Quantitative evaluation of rough roof of tunnels

**Fig 22:**
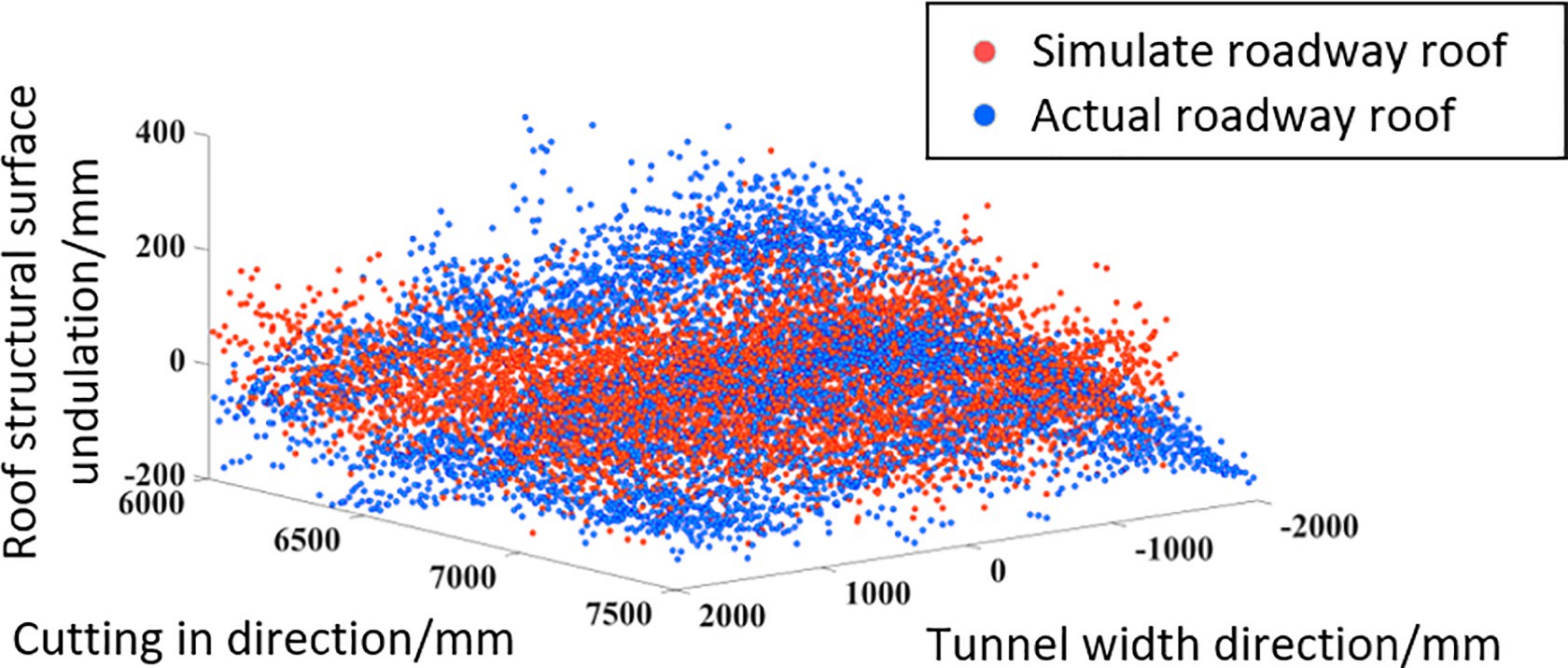
Comparison of roof point cloud data.

The fractal dimension D = 2.195 and the feature scale coefficient G = 10^-6^mm of the roof quantification evaluation method are determined based on the surface roughness of the roadway and the maximum distance between the peak and valley. Based on the above value parameters, simulated rough roof surface feature point data is obtained, and actual rough roof feature point data is obtained using the image grayscale mapping method. Express the feature points obtained by the above two methods in the image, as shown in Fig 22. From the diagram, it can be seen that the point cloud data in the diagram does not show a clear separation trend, and the actual roof and simulated roof point cloud data are well integrated.

Compare and analyze the surface characteristic parameters of simulated roof and actual rough roof using the above statistical evaluation methods, and conduct evaluation research on quantitative methods for rough roof. For the constructed roof feature point data, calculate the maximum range of rough surface peaks and valleys, standard deviation of rough surface height, skewness coefficient of rough surface wave peaks, and kurtosis coefficient of rough surface wave peaks between the simulated rough roof and the actual rough roof, respectively. The results are shown in Table 4.

**Table 4:** Statistical characteristic parameters of roof.

*Roof type*	*peak valley maximum range R/mm*	*height standard deviation σ*_*z*_ */mm*	*peak skewness coefficient S*	*peak state coefficient K*
*actual top plate*	600.7	87.59	0.1481	3.017
*simulate top plate*	608.4	66.12	0.1233	3.104

From Table 4, it can be seen that the three-dimensional morphology of the rough roof obtained by the quantitative evaluation method of the roof has significant statistical similarity with the three-dimensional morphology of the actual roof. In terms of peak valley range, the simulated and actual peak valley range of the roof are relatively close, with an error of only 1.3%, which is consistent with the performance within the rough scale range. In terms of height standard deviation, the actual roof height standard deviation is larger than the simulated roof height standard deviation, with a relative error of 24.5%, indicating that the actual roof morphology distribution has a greater degree of dispersion compared to the simulated roof. However, in terms of peak skewness coefficient and kurtosis coefficient, both peak skewness coefficients are close to 0, with a relative error of 16%. The peak kurtosis coefficients are both close to 3, with an error of only 2.9%, indicating that the surface morphology distribution characteristics of the two are very similar and have similar statistical characteristics of normal distribution.

**Fig 23:**
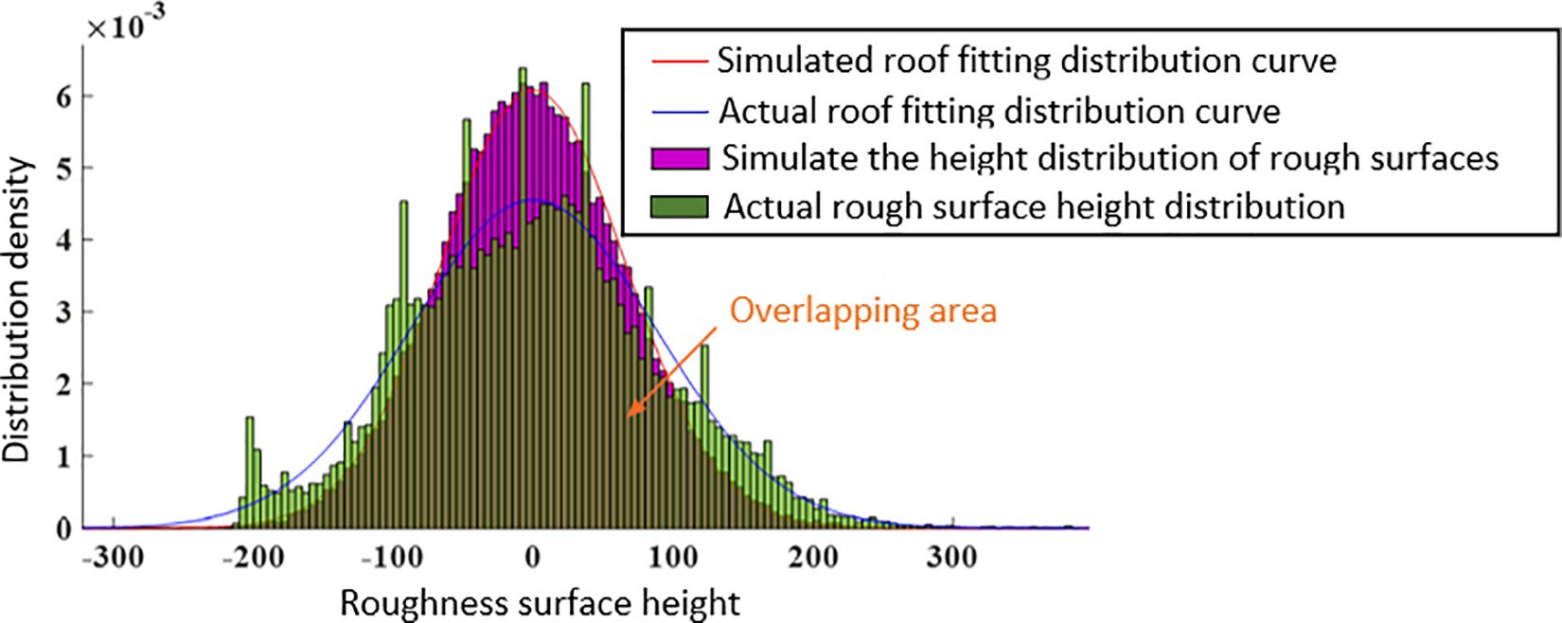
Distribution law of roof roughness plane.

The distribution density and fitted distribution curve of the actual roof and simulated roof are represented by histograms, as shown in Fig 23. According to the figure, it is found that the distribution trend of the two shows a normal distribution. The probability density of the distribution of the actual roof and simulated roof is fitted by the normal distribution probability density function. The specific probability density distribution function is as follows:

The fitting function for simulating the top plate is:

$$f(t)=\frac{1}{166.4}e^{-\frac{t^{2}}{8817.92}}$$ (24)

The fitting function for the actual roof is:

$$f(t)=\frac{1}{221.8}e^{-\frac{t^{2}}{15664.5}}$$ (25)

Due to the smaller peak to peak coefficient of the actual roof compared to the simulated roof, the height distribution of the simulated roof has a more obvious peak aggregation. However, the overall trend of the two distributions is basically the same, and there are also obvious overlapping areas in the distribution, reflecting the similarity between the two in terms of structural height fluctuations.

By using graphical comparison, statistical feature description, and distribution pattern comparison, it can be concluded that the simulated roof obtained through quantitative evaluation has significant similarity in surface morphology characteristics with the actual sampled roof. At the same time, statistical analysis shows that the morphology distribution patterns of the two are also very similar. Therefore, through comprehensive evaluation using multiple methods, it can be demonstrated that the quantitative evaluation method of rough roof morphology proposed in this article can effectively describe the characteristics of the roof, and has played a good quantitative role in the rough morphology of the roof after excavation.

## Conclusion

Based on the actual geological environment of the 122304 working face in Zhamei Tiebei Coal Mine, a quantitative study was conducted on the roof morphology of the fully mechanized roadway after cutting. The following conclusions have been drawn:

1. Based on the automatic cutting motion trajectory, cutting roof morphology, and the roughness of the cut roof in intelligent excavation, a quantitative evaluation method for the cut roof roughness is proposed.
2. Based on the actual surface characteristics of the roof, the height mapping processing of the grayscale image of the roadway roof was carried out, and the actual rough surface feature point cloud data of the roadway was obtained. The simulated roof generated by statistical and quantitative methods was compared and evaluated.
3. After statistical analysis, the relative errors between the maximum range of peaks and valleys, the peak skewness coefficient of height standard deviation, and the kurtosis coefficient of the actual roof were obtained to be 1.3%, 24.5%, 16%, and 2.9%, respectively. This overall indicates that the surface morphology distribution characteristics of the simulated roof and the actual roof are similar, verifying the correctness of the quantitative method proposed in this paper.

## Supporting information

S1 File (ZIP)
