# Supplementary material for: Analysis of the quality of tunnel roof topography by automatic cutting control under the coupling of multiple factors
Source: PLoS One. 2024 Mar 21;19(3):e0299805. doi: 10.1371/journal.pone.0299805 (PMC10956871; doi:10.1371/journal.pone.0299805)
Supplement: S1 File — (ZIP) [file pone.0299805.s001.zip › Supporting_Information_files/Fig10. Simulation results of residual between cutting rows in wide tunnels.docx]

% syms l0 dl0 h1 h2 h3 h4 l1 l2 l3 l4 dl3 theta1 theta2 xp yp zp

clc;clear;

l0 = 0;

l1 = 3000;

l2 = 700;

l3 = 2470;

l4 = 1775;

h1 = 1000;

h2 = 519;

h3 = 87;

h4 = 120;

dl3 = 0;

r1 = 416;

alpha = 83.5/180*pi;

beta = 73/180*pi;

n1 = 640;

n2 = 200;

% n1 = 360;

% n2 = 200;

theta0 = 0;

w = 0.016;

t = 0;

pd = zeros(4,1)

% theta1 = theta0+w*t;

theta2 = 41/180*pi;

theta1 = theta0

dl0 = 600;

dl3 = 0;

% syms l0 dl0 h1 h2 h3 h4 l1 l2 l3 l4 dl3 theta1 theta2 xp yp zp

%% 第一段

T0 = [1 0 0 0;0 1 0 l0;0 0 1 h1;0 0 0 1];

T1 = [cos(theta1) -sin(theta1) 0 0;sin(theta1) cos(theta1) 0 l1; 0 0 1 h2; 0 0 0 1];

T2 = [1 0 0 0; 0 cos(theta2) -sin(theta2) l2;0 sin(theta2) cos(theta2) 0;0 0 0 1];

T3 = [1 0 0 0;0 1 0 l3+dl3;0 0 1 h3; 0 0 0 1];

T4 = [1 0 0 0;0 1 0 l4;0 0 1 -h4;0 0 0 1];

T04 = T0*T1*T2*T3*T4;

% T04 = simplify(T04);

%T04 = double(T04)

% p4 = [xp yp zp 1]';

% p = T04*p4

% latex(p)

dl01 = 300;

dl02 = 300;

Poj = T04*[0 -r1 0 1]';

Po4 = T04*[0 0 0 1]';

Pc1 = T04*[0 -r1*(1-cos(alpha)) r1*sin(alpha) 1]';

Pc2 = T04*[0 -r1-n1 r1*sin(alpha)+(n1+r1*cos(alpha))/tan(beta) 1]';

Pc3 = T04*[0 -r1-n1-n2 r1*sin(alpha)+n2/tan(beta) 1]';

Pyoj = Poj(2);

Y = Pc1(2):1:Po4(2)

Z = (r1*sin(acos((Y-Poj(2))/r1))+Poj(3)).^((Y>=Pc1(2))&(Y<=Po4(2)))

plot(Y,Z)

hold on ;

Y1 = Pc2(2):1:Pc1(2)

Z1 = ((Pc2(3)-Pc1(3))/(Pc2(2)-Pc1(2))*(Y1-Pc2(2))+Pc2(3)).^((Y1>=Pc2(2))&(Y1<=Pc1(2)))

plot(Y1,Z1)

%% 第二段

T01 = [1 0 0 0;0 1 0 l0+dl0;0 0 1 h1;0 0 0 1];

T11 = [cos(theta1) -sin(theta1) 0 0;sin(theta1) cos(theta1) 0 l1; 0 0 1 h2; 0 0 0 1];

T21 = [1 0 0 0; 0 cos(theta2) -sin(theta2) l2;0 sin(theta2) cos(theta2) 0;0 0 0 1];

T31 = [1 0 0 0;0 1 0 l3+dl3;0 0 1 h3; 0 0 0 1];

T41 = [1 0 0 0;0 1 0 l4;0 0 1 -h4;0 0 0 1];

T041 = T01*T11*T21*T31*T41;

Poj1 = T041*[0 -r1 0 1]';

Po41 = T041*[0 0 0 1]';

Pc11 = T041*[0 -r1*(1-cos(alpha)) r1*sin(alpha) 1]';

Pc21 = T041*[0 -r1-n1 r1*sin(alpha)+(n1+r1*cos(alpha))/tan(beta) 1]';

Pc31 = T041*[0 -r1-n1-n2 r1*sin(alpha)+n2/tan(beta) 1]';

Pyoj = Poj(2);

Y01 = Pc11(2):1:Po41(2)

Z01 = (r1*sin(acos((Y01-Poj1(2))/r1))+Poj1(3)).^((Y01>=Pc11(2))&(Y01<=Po41(2)))

plot(Y01,Z01)

hold on ;

Y11 = Pc21(2):1:Pc11(2)

Z11 = ((Pc21(3)-Pc11(3))/(Pc21(2)-Pc11(2))*(Y11-Pc21(2))+Pc21(3)).^((Y11>=Pc21(2))&(Y11<=Pc11(2)))

plot(Y11,Z11)

%% 第三段

T02 = [1 0 0 0;0 1 0 l0+1000;0 0 1 h1;0 0 0 1];

T12 = [cos(theta1) -sin(theta1) 0 0;sin(theta1) cos(theta1) 0 l1; 0 0 1 h2; 0 0 0 1];

T22 = [1 0 0 0; 0 cos(theta2) -sin(theta2) l2;0 sin(theta2) cos(theta2) 0;0 0 0 1];

T32 = [1 0 0 0;0 1 0 l3+dl3;0 0 1 h3; 0 0 0 1];

T42 = [1 0 0 0;0 1 0 l4;0 0 1 -h4;0 0 0 1];

T042 = T02*T12*T22*T32*T42;

Poj2 = T042*[0 -r1 0 1]';

Po42 = T042*[0 0 0 1]';

Pc12 = T042*[0 -r1*(1-cos(alpha)) r1*sin(alpha) 1]';

Pc22 = T042*[0 -r1-n1 r1*sin(alpha)+(n1+r1*cos(alpha))/tan(beta) 1]';

Pc32 = T042*[0 -r1-n1-n2 r1*sin(alpha)+n2/tan(beta) 1]';

Pyoj = Poj(2);

Y02 = Pc12(2):1:Po42(2)

Z02 = (r1*sin(acos((Y02-Poj2(2))/r1))+Poj2(3)).^((Y02>=Pc12(2))&(Y02<=Po42(2)))

plot(Y02,Z02)

hold on ;

Y12 = Pc22(2):1:Pc12(2)

Z12 = ((Pc22(3)-Pc12(3))/(Pc22(2)-Pc12(2))*(Y12-Pc22(2))+Pc22(3)).^((Y12>=Pc22(2))&(Y12<=Pc12(2)))

plot(Y12,Z12)

%% 求交点

syms Y

f1 = r1*sin(acos((Y-Poj(2))/r1))+Poj(3)-((Pc21(3)-Pc11(3))/(Pc21(2)-Pc11(2))*(Y-Pc21(2))+Pc21(3));

s1 = double(solve(f1,Y))

s1 = s1(1)

f2 = r1*sin(acos((Y-Poj1(2))/r1))+Poj1(3)-(r1*sin(acos((Y-Poj2(2))/r1))+Poj2(3));

s2 = double(solve(f2,Y))

plot(s1,(Pc21(3)-Pc11(3))/(Pc21(2)-Pc11(2))*(s1-Pc21(2))+Pc21(3),'v')

plot(s2,r1*sin(acos((s2-Poj1(2))/r1))+Poj1(3),'o')

r1*sin(acos((s1-Poj(2))/r1))+Poj(3)

r1*sin(acos((s1-Poj1(2))/r1))+Poj1(3)

%% 函数连续表达

Y = 6300:2:Po41(2);

Z = (r1*sin(acos((Y-Poj(2))/r1))+Poj(3)).^((Y>=Pc1(2))&(Y<=s1))+((Pc2(3)-Pc1(3))/(Pc2(2)-Pc1(2))*(Y-Pc2(2))+Pc2(3)).^((Y>=Pc2(2))&(Y<=Pc1(2)))...

+(r1*sin(acos((Y-Poj1(2))/r1))+Poj1(3)).^((Y>=Pc11(2))&(Y<=s2))+((Pc21(3)-Pc11(3))/(Pc21(2)-Pc11(2))*(Y-Pc21(2))+Pc21(3)).^((Y>=s1)&(Y<=Pc11(2)))...

+(r1*sin(acos((Y-Poj2(2))/r1))+Poj2(3)).^((Y>=s2)&(Y<=Po42(2)))

plot(Y,Z-220,'o-','linewidth',3)

set(gcf,'color','w')

grid off

set(gca,'FontName','Times New Roman','FontSize',36)

%legend('改进离散法','全离散法','半离散法','Location','NorthEast','FontName','Songti');

xlabel('截割进给方向/mm','FontName','宋体','FontSize',48);

ylabel('截割头运动轮廓/mm','FontName','宋体','FontSize',48)

%%

%[yz,zz] = meshgrid(Y,Z);

n=1000;

theta00=(0:n)/n*2*pi;

%theta00

X = Y'*sin(theta00);

% X1 = Y'*sin(theta00)+1000;

%

% X2 = Y'*sin(theta00)-1000;

Y = Y'*cos(theta00);

Ys = size(Y)

Z = max(max(Z))-Z'*ones(1,n+1);

%Z = Z'*ones(1,n+1)+rand(Ys(1),n+1)*100.*(rand(Ys(1),n+1)>0.9);

figure(4)

surfc(X,Y,Z)

% hold on;

% surfc(XG,YG,ZG)

% surfc(X,Y,Z)

shading interp

colormap('jet')

set(gcf,'color','w')

grid off

set(gca,'FontName','Times New Roman','FontSize',36,'FontWeight','bold')

%legend('改进离散法','全离散法','半离散法','Location','NorthEast','FontName','Songti');

xlabel('巷道宽度方向/mm','FontName','宋体','FontWeight','bold','FontSize',48);

ylabel('截割进给方向/mm','FontName','宋体','FontWeight','bold','FontSize',48)

zlabel('截割行间残留/mm','FontName','宋体','FontWeight','bold','FontSize',48);

xlim([-2000 2000])

ylim([6000 7650])

% zlim([3000 4600])

view(0,90);

% X=x'*sin(theta);

% Y=x'*cos(theta);

% Z=z'*ones(1,n+1);

% mesh(X,Y,Z)

%

% figure(2)

% mesh(X,Y,Z)

%% 分形

figure(3)

L = 256;

Ls = 1;

r = 1.5;

M = 3;

nmax = int8(log(L/Ls)/log(r))

z1 = 0;

z2 = 0;

X1 = linspace(0,4000,1001);

Y1 = linspace(0,8000,613);

D = 2.195;

G = 10^-6;

[x,y]=meshgrid(X1,Y1);

for m = 1:1:M

for n = 1:1:14

A =rand.*2*pi;

z1 = z1+(cos(A)-cos(2*pi*r^n.*(x.^2+y.^2).^0.5./L.*cos(atan(y./x)-pi*m/M)+A));

end

z2 = z2+z1;

end

z2 = z2*L*(G/L)^(D-2);

surfc(x,y,z2)

hold on

shading interp

%meshz(x,y,z2)

colormap('jet')

set(gcf,'color','w')

set(gca, 'LineWidth',1.5)

set(gca,'FontName','Times New Roman','FontSize',36,'FontWeight','bold')

%legend('改进离散法','全离散法','半离散法','Location','NorthEast','FontName','Songti');

xlabel('巷道宽度方向/m','FontName','宋体','FontWeight','bold','FontSize',48,'Rotation',20);

ylabel('巷道长度方向/m','FontName','宋体','FontWeight','bold','FontSize',48,'Rotation',-25);

title('D=2.2,G=10^{-6}','FontName','宋体','FontSize',48,'FontWeight','bold');

%title('截割宽度352mm,时间周期离散数m=100','FontName','SongTi','FontSize',48,'FontWeight','bold')

view(140,60)

% y1 = reshape(y,[13*13,1])

% x1 = reshape(x,[13*13,1])

% z3 = reshape(z2,[13*13,1])

% z3 = mean(z2)

% z3 = mean(z3(2:end))

% z3_ = z3*ones(100,100)

% surfc(x,y,z3_)

%colormap('Gray')

% z4 = reshape(z2,[100*100,1])

%meshz(x,y,z2)

%%

Zz = Z+z2;

%%

figure(2)

surfc(X,Y,Zz)

shading interp

colormap('jet')

set(gcf,'color','w')

grid off

set(gca,'FontName','Times New Roman','FontSize',36,'FontWeight','bold')

%legend('改进离散法','全离散法','半离散法','Location','NorthEast','FontName','Songti');

xlabel('巷道宽度方向/mm','FontName','宋体','FontWeight','bold','FontSize',48);

ylabel('截割进给方向/mm','FontName','宋体','FontWeight','bold','FontSize',48)

zlabel('模拟顶板结构面起伏/mm','FontName','宋体','FontWeight','bold','FontSize',48);

zlim([-200,400])

xlim([-2000 2000])

ylim([6000 7500])

% zlim([4000 4900])

view(145,50);

%%

x12 = reshape(X,[613*1001,1])

x12 = x12(2:10:end);

y12 = reshape(Y,[613*1001,1])

y12 = y12(2:10:end);

Zzz = reshape(Zz,[613*1001,1]);

Zzz1 = Zzz(2:10:end)

Zzz2 = Zzz1-mean(Zzz1)

figure(10)

scatter3(x12,y12,Zzz2,'r')

hold on

%

x1 = linspace(0,4000,294);

y1 = linspace(6000,7500,207);

[X1,Y1] = meshgrid(x1,y1);

x11 = reshape(X1,[294*207,1]);

y11 = reshape(Y1,[294*207,1]);

f = imread('dingban2.png');% 从目标路径读入图像

gray_f = rgb2gray(f); % 灰度化处理

gray_f = gray_f(2:end-1,2:end-8);

gray_f1 = reshape(gray_f,[294*207,1]);

gray_f1 = double(gray_f1)

gray_max = max(gray_f1)

gray_min = min(gray_f1)

gray_mean = mean(gray_f1)

gray_f1 = (gray_f1-gray_mean)/(gray_max-gray_min)*600

gray_f2 = reshape(gray_f1,[207,294])

%gray_f = gray_f(1:4:end,1:4:end)

scatter3(x11(1:10:end)-2000,y11(1:10:end),gray_f1(1:10:end),'b')

zlim([-200,400])

xlim([-2000 2000])

ylim([6000 7500])

% zlim([4000 4900])

view(145,50);

set(gcf,'color','w')

grid off

set(gca,'FontName','Times New Roman','FontSize',36,'FontWeight','bold')

%legend('改进离散法','全离散法','半离散法','Location','NorthEast','FontName','Songti');

xlabel('巷道宽度方向/mm','FontName','宋体','FontWeight','bold','FontSize',48);

ylabel('截割进给方向/mm','FontName','宋体','FontWeight','bold','FontSize',48)

zlabel('模拟顶板结构面起伏/mm','FontName','宋体','FontWeight','bold','FontSize',48);

legend('模拟顶板','实际巷道顶板','FontName','宋体','FontWeight','bold','FontSize',48)

%%

Xtmax = max(X)

Xtmin = min(X)

Xtmean = mean(X)

Xt = (X-Xtmin)/(Xtmax-Xtmin)*6000

Ytmax = max(Y)

Ytmin = min(Y)

Ytmean = mean(Y)

Yt = (Y-Ytmin)/(Ytmax-Ytmin)*6000

Zz(1)=205;

Zztmax = max(Zz)

Zztmin = min(Zz)

Zztmean = mean(Zz)

Zzt = (Zz-Zztmin)/(Zztmax-Zztmin)*255

imshow(Zz)

%%

% z4 = reshape(z2,[100*100,1])

%%

%极差

Zzz = reshape(Zz,[613*1001,1]);

Zzz =Zzz(2:end);

Zzz1 = Zzz(1:10:end)

Zzz2 = Zzz1-mean(Zzz1)

zrange=range(Zzz)%极差

%方差

zvar=var(Zzz)%方差

%标准差

zstd=std(Zzz)%标准差

%偏度

zske=skewness(Zzz,0)%偏度

%峰度

zkur=kurtosis(Zzz,0)%峰度
