# Supplementary material for: Analysis of the quality of tunnel roof topography by automatic cutting control under the coupling of multiple factors
Source: PLoS One. 2024 Mar 21;19(3):e0299805. doi: 10.1371/journal.pone.0299805 (PMC10956871; doi:10.1371/journal.pone.0299805)
Supplement: S1 File — (ZIP) [file pone.0299805.s001.zip › Supporting_Information_files/Fig13. 3D Fractal Surface Morphologyú¿aú⌐.docx]

clc;

clear;

L = 256;

Ls = 1;

r = 1.5;

M = 3;

nmax = int8(log(L/Ls)/log(r))

z1 = 0;

z2 = 0;

X = linspace(0,4000,100);

Y = linspace(0,8000,100);

D = 2.2;

G = 10^-6;

[x,y]=meshgrid(X,Y);

for m = 1:1:M

for n = 1:1:14

A =rand.*2*pi;

z1 = z1+(cos(A)-cos(2*pi*r^n.*(x.^2+y.^2).^0.5./L.*cos(atan(y./x)-pi*m/M)+A));

end

z2 = z2+z1;

end

z2 = z2*L*(G/L)^(D-2);

surfc(x,y,z2)

hold on

shading interp

%meshz(x,y,z2)

colormap('jet')

set(gcf,'color','w')

set(gca, 'LineWidth',1.5)

set(gca,'FontName','Times New Roman','FontSize',36,'FontWeight','bold')

%legend('改进离散法','全离散法','半离散法','Location','NorthEast','FontName','Songti');

xlabel('巷道宽度方向/m','FontName','宋体','FontWeight','bold','FontSize',48,'Rotation',20);

ylabel('巷道长度方向/m','FontName','宋体','FontWeight','bold','FontSize',48,'Rotation',-25);

title('D=2.2,G=10^{-6}','FontName','宋体','FontSize',48,'FontWeight','bold');

%title('截割宽度352mm,时间周期离散数m=100','FontName','SongTi','FontSize',48,'FontWeight','bold')

view(140,60)
