# Supplementary material for: Analysis of the quality of tunnel roof topography by automatic cutting control under the coupling of multiple factors
Source: PLoS One. 2024 Mar 21;19(3):e0299805. doi: 10.1371/journal.pone.0299805 (PMC10956871; doi:10.1371/journal.pone.0299805)
Supplement: S1 File — (ZIP) [file pone.0299805.s001.zip › Supporting_Information_files/Fig20. Gray scale and histogram of roadway roof.docx]

f = imread('dingban.png');

% Gray histogram Test Function

%% 1.读入图像：打开指定路径图片

f = imread('dingban.png'); % 读图

%% 2.灰度化

gray_f = rgb2gray(f); % 灰度化处理

%% 3.保存图片

%imwrite(gray_f,'E:\matlab\2020a\cx\fuzhuang\paper\Gray histogram\pic\11.jpg');

%% 4.读取图片信息（可要可不要）

%info = imfinfo('E:\matlab\2020a\cx\fuzhuang\paper\Gray histogram\pic\11.jpg');

%% 5.histeq均衡化处理（默认n=64）

R1 = histeq(gray_f); % 使用直方图均衡增强灰度图像的对比度

%% 6.adapthisteq均衡化处理

R2 = adapthisteq(gray_f); % adapthisteq均衡后的直方图

%% 7.imadjust灰度范围调整

R3 = imadjust(gray_f,[0.2 0.5],[0 1]); % 调整灰度值

%% 8.效果图显示

figure,

subplot(231);imshow(f);title('原图');

subplot(232);imshow(gray_f);title('灰度图像');

subplot(233);imshow(R1);title('histeq均衡化结果图（默认n=64）');

subplot(234);imshow(R2);title('adapthisteq均衡化结果图');

subplot(235);imshow(R3);title('imadjust灰度范围调整结果图');

%% 9.直方图显示

figure,

subplot(221);imhist(gray_f);title('Gray Histogram'); % 绘制并显示灰度直方图

subplot(222);imhist(R1);title('histeq均衡化直方图'); % 显示histeq均衡化后的直方图

subplot(223);imhist(R2);title('adapthisteq均衡化直方图'); % 显示adapthisteq均衡化后的直方图

subplot(224);imhist(R3);title('imadjust灰度范围调整后直方图'); % 显示imadjust灰度范围调整后的直方图
