# Supplementary material for: Analysis of the quality of tunnel roof topography by automatic cutting control under the coupling of multiple factors
Source: PLoS One. 2024 Mar 21;19(3):e0299805. doi: 10.1371/journal.pone.0299805 (PMC10956871; doi:10.1371/journal.pone.0299805)
Supplement: S1 File — (ZIP) [file pone.0299805.s001.zip › Supporting_Information_files/Fig21. Real Coal-rock Rough Surface 3D Surface.docx]

clc;

clear;

L = 256;

Ls = 1;

r = 1.5;

M = 3;

nmax = int8(log(L/Ls)/log(r))

z1 = 0;

z2 = 0;

X = linspace(0,4000,100);

Y = linspace(0,8000,100);

D = 2.2;

G = 10^-6;

[x,y]=meshgrid(X,Y);

for m = 1:1:M

for n = 1:1:14

A =rand.*2*pi;

z1 = z1+(cos(A)-cos(2*pi*r^n.*(x.^2+y.^2).^0.5./L.*cos(atan(y./x)-pi*m/M)+A));

end

z2 = z2+z1;

end

z2 = z2*L*(G/L)^(D-2);

surfc(x,y,z2)

hold on

shading interp

%meshz(x,y,z2)

colormap('jet')

set(gcf,'color','w')

set(gca, 'LineWidth',1.5)

set(gca,'FontName','Times New Roman','FontSize',36,'FontWeight','bold')

%legend('改进离散法','全离散法','半离散法','Location','NorthEast','FontName','Songti');

xlabel('巷道宽度方向/m','FontName','宋体','FontWeight','bold','FontSize',48,'Rotation',20);

ylabel('巷道长度方向/m','FontName','宋体','FontWeight','bold','FontSize',48,'Rotation',-25);

title('D=2.2,G=10^{-6}','FontName','宋体','FontSize',48,'FontWeight','bold');

%title('截割宽度352mm,时间周期离散数m=100','FontName','SongTi','FontSize',48,'FontWeight','bold')

view(140,60)

% y1 = reshape(y,[13*13,1])

% x1 = reshape(x,[13*13,1])

% z3 = reshape(z2,[13*13,1])

z3 = mean(z2)

z3 = mean(z3(2:end))

z3_ = z3*ones(100,100)

surfc(x,y,z3_)

%colormap('Gray')

z4 = reshape(z2,[100*100,1])

%%

%极差

z4 = z4(2:end)

zrange=range(z4)%极差

%方差

zvar=var(z4)%方差

%标准差

zstd=std(z4)%标准差

%偏度

zske=skewness(z4,0)%偏度

%峰度

zkur=kurtosis(z4,0)%峰度

%%

x1 = linspace(0,4000,294);

y1 = linspace(6000,7500,207);

[X1,Y1] = meshgrid(x1,y1);

x11 = reshape(X1,[294*207,1]);

y11 = reshape(Y1,[294*207,1]);

f = imread('dingban2.png');% 从目标路径读入图像

gray_f = rgb2gray(f); % 灰度化处理

gray_f = gray_f(2:end-1,2:end-8);

gray_f1 = reshape(gray_f,[294*207,1]);

gray_f1 = double(gray_f1)

gray_max = max(gray_f1)

gray_min = min(gray_f1)

gray_mean = mean(gray_f1)

gray_f1 = (gray_f1-gray_mean)/(gray_max-gray_min)*600

gray_f2 = reshape(gray_f1,[207,294])

%gray_f = gray_f(1:4:end,1:4:end)

% figure(6)

% scatter3(x11(1:10:end),y11(1:10:end),gray_f1(1:10:end))

hold on

surf(X1,Y1,gray_f2)

view(145,50);

shading interp

colormap('jet')

set(gcf,'color','w')

grid off

set(gca,'FontName','Times New Roman','FontSize',36,'FontWeight','bold')

%legend('改进离散法','全离散法','半离散法','Location','NorthEast','FontName','Songti');

xlabel('巷道宽度方向/mm','FontName','宋体','FontWeight','bold','FontSize',48);

ylabel('截割进给方向/mm','FontName','宋体','FontWeight','bold','FontSize',48)

zlabel('实际顶板结构面起伏/mm','FontName','宋体','FontWeight','bold','FontSize',48);

figure(4)

subplot(121);imshow(gray_f);

set(gcf,'color','w')

set(gca, 'LineWidth',1.5)

set(gca,'FontName','Times New Roman','FontSize',36,'FontWeight','bold')

title('灰度图像','FontName','宋体','FontWeight','bold','FontSize',48);

subplot(122);

imhist( gray_f);

set(gca, 'LineWidth',1.5)

set(gca,'FontName','Times New Roman','FontSize',36,'FontWeight','bold');

title('灰度直方图','FontName','宋体','FontWeight','bold','FontSize',48);

grange=range(gray_f1)%极差

%方差

gvar=var(gray_f1)%方差

%标准差

gstd=std(gray_f1)%标准差

%偏度

gske=skewness(gray_f1,0)%偏度

%峰度

gkur=kurtosis(gray_f1,0)%峰度

% title('Gray Histogram'); % 绘制并显示灰度直方图

%%

clc;

clear;

D = 2.2

G = (1:0.2:10)*10^-6;

L = 1;

r = 1.5;

oo = sqrt(G.^(2*(D-2))./(2*log(r)).*(1./(6-2*D)).*L.^(6-2*D))

plot(D,oo,'o')
